# Secure and Intelligent Low-Altitude Infrastructures: Synergistic Integration of IoT Networks, AI Decision-Making and Blockchain Trust Mechanisms

**DOI:** 10.3390/s25216751

**Published:** 2025-11-04

**Authors:** Yuwen Ye, Xirun Min, Xiangwen Liu, Xiangyi Chen, Kefan Cao, S. M. Ruhul Kabir Howlader, Xiao Chen

**Affiliations:** 1 School of Computer Science and Communication Engineering, Jiangsu University, Zhenjiang 212013, China; yuwen.ye@stmail.ujs.edu.cn (Y.Y.); xirun.min@stmail.ujs.edu.cn (X.M.); 2 School of Computing, Newcastle University, Newcastle NE1 7RU, UK; k.cao7@newcastle.ac.uk; 3 School of Computing Science and Mathematical Sciences, University of Leicester, Leicester LE1 7RH, UK; smrkh1@leicester.ac.uk (S.M.R.K.H.); xiao.chen@leicester.ac.uk (X.C.)

**Keywords:** low-altitude economy, unmanned aerial vehicles, Internet of Things, blockchain, artificial intelligence

## Abstract

The low-altitude economy (LAE), encompassing urban air mobility, drone logistics and sub 3000 m aerial surveillance, demands secure, intelligent infrastructures to manage increasingly complex, multi-stakeholder operations. This survey evaluates the integration of Internet of Things (IoT) networks, artificial intelligence (AI) decision-making and blockchain trust mechanisms as foundational enablers for next-generation LAE ecosystems. IoT sensor arrays deployed at ground stations, unmanned aerial vehicles (UAVs) and vertiports form a real-time data fabric that records variables from air traffic density to environmental parameters. These continuous data streams empower AI models ranging from predictive analytics and computer vision (CV) to multi-agent reinforcement learning (MARL) and large language model (LLM) reasoning to optimize flight paths, identify anomalies and coordinate swarm behaviors autonomously. In parallel, blockchain architectures furnish immutable audit trails for regulatory compliance, support secure device authentication via decentralized identifiers (DIDs) and automate contractual exchanges for services such as airspace leasing or payload delivery. By examining current research and practical deployments, this review demonstrates how the synergistic application of IoT, AI and blockchain can bolster operational efficiency, resilience and trustworthiness across the LAE landscape.

## 1. Introduction

### 1.1. Broad Context: The Emergence and Significance of the Low-Altitude Economy (LAE)

The emergence of novel aviation technologies, coupled with a burgeoning demand for innovative solutions, has catalyzed the rise of the LAE, which is projected to become a multi-billion-dollar industry. The LAE comprises economic activities and services conducted within airspace typically below 3000 m, propelled predominantly by technological advancements in Unmanned Aerial Vehicles (UAVs), electric Vertical Take-off and Landing (eVTOL), and sophisticated autonomous aviation systems [1,2]. It is essential to recognize that the LAE extends beyond the aircraft themselves to encompass the entire operational ecosystem, including ground support systems, Air Traffic Management (ATM) infrastructure, and the associated regulatory frameworks.

The applications of the LAE are extensive, spanning Urban Air Mobility (UAM), often termed “air taxis” [3]; logistical transport and last-mile delivery of cargo and medical supplies [4]; smart agriculture for crop monitoring and precision spraying [5,6]; inspection of infrastructure assets [7,8]; disaster response and damage assessment [9] and environmental monitoring [10].

The development of the LAE represents not merely an expansion of the aviation industry but an economic transformation poised to reshape urban-rural connectivity, revolutionize supply chains, and redefine service delivery models. This transformation is propelled by the confluence of key technologies, including UAVs, Artificial Intelligence (AI) and advanced communications networks, and the societal need to address challenges such as urban congestion, the demand for greater logistical efficiency, the servicing of remote regions, and the enhancement of public safety and environmental management. Spanning applications from sophisticated UAV systems to automated agricultural solutions, the LAE is catalyzing cross-domain innovation and the emergence of new economic paradigms, fundamentally reshaping societal modes of mobility, logistics, and human–environment interaction.

### 1.2. Problem Statement: Critical Challenges Hindering Large-Scale LAE Deployment

Despite its considerable potential, the widespread adoption of the LAE confronts multifaceted challenges centered on ensuring safety, advancing autonomy, and building stakeholder trust. These are prerequisites for the effective management of a diverse and complex low-altitude airspace.

Safety challenges are paramount. Airspace security risks include unauthorized drone incursions and attacks on ground infrastructure [11,12], while cybersecurity threats include compromise of control systems through hacking, data breaches leading to the loss of sensitive information, GPS spoofing, and Denial-of-Service (DoS) attacks targeting Ground Control Stations (GCS) [13]. Furthermore, the low operational altitudes and critical reliance on wireless communication render these aircraft particularly susceptible to ground-based cyberattacks [14].

The challenge of achieving advanced intelligence stems from the inherent complexity of the low-altitude operational environment. This requires intelligent systems capable of managing high-density air traffic and enabling unmanned systems to make autonomous decisions under dynamic conditions [15]. In addition, predictive capabilities are essential to forecast airspace congestion, assess meteorological risks, and optimize resource allocation. However, deploying large-scale AI models is hindered by computational constraints and the difficulty of adapting laboratory-trained models to real-world complexities [16].

Establishing trust is a complex challenge influenced by public and regulatory acceptance [17]. As this review centers on the enabling technologies, the discussion of trust will concentrate on the technical dimensions, particularly those related to data security, privacy, and the verifiability of autonomous operations. Privacy concerns are particularly prominent, driven by the potential for widespread surveillance by camera-equipped drones and large-scale data collection. The operational opacity of autonomous systems can foster apprehension, making verifiable identities for agents like UAVs and AI units critical for regulatory compliance and public confidence.

These three challenges are not isolated. Rather, they are intricately interwoven, forming a complex network of interdependencies that demands a systematic and integrated response. Only through the synergistic development and mutual reinforcement of these three pillars can the comprehensive implementation of a safe, intelligent, and trustworthy LAE be achieved.

### 1.3. Proposed Solution: Synergistic Integration of IoT, AI, and Blockchain

The core thesis of this review is that the synergistic integration of the Internet of Things (IoT), AI, and blockchain provide a robust and systematic foundation to address the interdependent challenges of safety, intelligence, and trust within the LAE. This integrated framework enables the construction of its next-generation infrastructure by assigning distinct but deeply interconnected roles to each technological pillar.

In this paradigm, IoT serves as the foundational perception layer, acting as the distributed sensory system of the LAE. AI functions as a cognitive engine, responsible for intelligent analysis and decision-making. Blockchain provides the indispensable trust layer, serving as a decentralized and immutable ledger for verification and transaction automation. The transformative power lies not in the technologies themselves, but in their synergy: IoT provides the data, AI provides the intelligence, and blockchain provides the trust. The detailed rationale, recent advances, and internal components of these technologies will be systematically reviewed in Section 3.

### 1.4. Contribution and Scope of the Review

This paper presents a dual contribution: it is first a systematic survey of state-of-the-art research on the application of IoT networks, AI decision-making, and blockchain-based trust mechanisms to low-altitude infrastructure. Second, based on this synthesis, it proposes a novel conceptual architecture—a holistic, layered framework designed to integrate these disparate technologies. To ensure a comprehensive coverage of the relevant literature, publications were retrieved from major academic databases and sources, including Web of Science, Scopus, Google Scholar, and the preprint repository arXiv. Keyword combinations such as “low-altitude infrastructure”, “UAV IoT” and “blockchain airspace” were employed during the search process. A distinctive feature of this research is its emphasis on the synergistic effects emerging from the deep fusion of these three technological pillars, moving beyond a single-technology perspective to focus on the holistic enabling infrastructure for the entire LAE. Existing literature typically focuses on integrating only two of these technologies or analyzes components in isolation [18]. While these dual-technology approaches have proven effective within their specific contexts, they often leave critical gaps; for example, an IoT-AI system may lack verifiable trust, while an IoT-Blockchain system may lack advanced intelligence.

More recently, a few pioneering studies have begun to explore the integration of all three pillars, providing preliminary evidence of their combined potential. However, these efforts are often application-specific and do not yet offer a comprehensive, scalable architectural blueprint. To bridge this gap, we summarize and compare these pioneering works in Table 1.

As evidenced by Table 1, while the feasibility of combining IoT, AI, and blockchain is being established, a significant research gap remains for a holistic, systemic framework that can guide the development of a truly secure and intelligent LAE. Recognizing the real-world demand for such an infrastructure and the current lack of a guiding theoretical model, this review proposes a conceptual architecture and aims to provide a foundational blueprint that is broadly applicable across various LAE scenarios.

To bridge this conceptual gap, it is essential to move beyond isolated technological discussions and examine how their integration fundamentally redefines system capabilities. The core thesis is that the true transformative power lies not in the individual technologies but in their deep, synergistic integration, which creates a system far more capable than the sum of its parts. Specifically, this combination delivers three critical enhancements:

(1) **Trusted Intelligence:** Blockchain provides a tamper-proof foundation for data collected by IoT devices. This ensures that AI models are trained and operate on data with verifiable integrity, addressing the “Garbage In, Garbage Out” problem and making AI decisions more reliable and auditable.
(2) **Intelligent Trust:** AI algorithms can enhance the blockchain layer itself by, for example, detecting anomalous transaction patterns or optimizing consensus mechanisms for resource-constrained IoT devices [23]. This makes the trust layer more adaptive and efficient.
(3) **Automated, Verifiable Operations:** The synergy enables a closed-loop system where IoT devices capture real-world events, AI makes optimized decisions, and blockchain-based smart contracts autonomously execute and verify these actions (e.g., flight path authorization, automated payments upon delivery) in a decentralized and trustworthy manner.

To guide this survey and structure our analysis, we formulate the following Research Questions (RQs):

- ***RQ1:** How can the IoT perception layer enhance situational awareness and data acquisition in low-altitude environments?*
  To answer this question, the paper provides a detailed technical review in Section 3.1, “IoT Perception Layer”. This section examines the core components that constitute the data acquisition foundation by first reviewing “Low-Altitude Aerial Platforms and Onboard Sensor Suites” (Section 3.1.1), which details various UAV types and sensors like LiDAR and RGB cameras. It then reviews the “Ground-Based Infrastructure, Sensor Networks and Communication Links” (Section 3.1.2), explaining how elements like vertiports, ground sensors, and communication networks work together to create a comprehensive perception network.
- ***RQ2:** What role do AI decision-making and analysis layers play in enabling autonomous operations and efficient coordination?*
  To answer this question, the paper dedicates Section 3.2, “AI Decision-Making and Analysis Layer”, to explaining AI’s role as the system’s “cognitive engine”. This section details how AI enables autonomy and coordination by reviewing the specific applications of key AI branches. It examines “CV for Real-Time Perception” (Section 3.2.1), “RL for Autonomous Path Planning and Control” (Section 3.2.2), “Predictive Analytics and Anomaly Detection” (Section 3.2.3), and the “Emerging Role of LLMs” (Section 3.2.4) in transforming raw data into intelligent, autonomous actions.
- ***RQ3:** How can blockchain mechanisms be applied to guarantee trust, ensure regulatory compliance, and facilitate secure transactions?*
  To answer this question, the paper provides a detailed review in Section 3.3, “Blockchain Trust and Traceability Layer”. This section explains how blockchain provides a “solid foundation of trust” by examining its three core components. It details the application of “Decentralized Identity (DID)” (Section 3.3.1) for secure entity verification, “Immutable Audit Trails” (Section 3.3.2) for regulatory compliance and incident investigation, and “Smart Contracts for Automation” (Section 3.3.3) for facilitating secure, automated transactions and agreements.
- ***RQ4:** What is the potential value and applicative role of the synergistic integration of IoT, AI, and blockchain in typical LAE scenarios?*
  To answer this question, the paper dedicates Section 4, “Typical Application Scenarios”, to illustrating the potential value and applicative role of the integrated framework. This section analyzes the core challenges in scenarios such as urban logistics, UAM, and precision agriculture, and demonstrates how the synergistic application of IoT, AI, and blockchain offers a conceptual blueprint for addressing these issues.
- ***RQ5:** What are the key open research challenges that must be addressed to realize a fully integrated, secure, and intelligent LAE infrastructure?*
  To address this question, Section 5, entitled “Challenges and Future Directions,” provides a comprehensive analysis of the major barriers to large-scale deployment and explores corresponding research opportunities. The discussion is structured around four complementary perspectives: “Technology Integration and Standardization” (Section 5.1), “Data Privacy and Security Risks” (Section 5.2), “Computing Resources and Real-time Constraints” (Section 5.3), and “Legal, Regulatory, and Ethical Frameworks” (Section 5.4). Each perspective identifies critical issues and outlines future research directions to advance the development of intelligent low-altitude infrastructure.

### 1.5. Paper Organization

The remainder of this paper is structured as follows:

Section 2 proposes a layered architectural model for a safe and intelligent LAE infrastructure, introducing each functional layer and its methods of interaction;

Section 3 provides a detailed review of the three core technologies: the IoT Perception Layer, the AI Decision-Making and Analysis Layer, and the Blockchain Trust and Traceability Layer;

Section 4 demonstrates the practical deployment of this integrated infrastructure by examining typical scenarios, including urban logistics, UAM with smart surveillance, and Precision Agriculture;

Finally, Section 5 summarizes the main findings, presents key challenges and future research directions, and emphasizes the significance of the deep integration of IoT, AI, and Blockchain for the future of the LAE.

## 2. Overall Architecture of Secure and Intelligent Low-Altitude Infrastructures

### 2.1. Introduction to the Architecture: The Imperative for a Well-Defined Architecture

The successful implementation of LAE is contingent on a robust infrastructure. Given that LAE systems inherently involve a multitude of heterogeneous components, including aerial platforms, GCS, sensor networks, and human stakeholders, a layered architecture is indispensable. Such a framework provides a structured approach to managing complexity, ensures interoperability among disparate systems, facilitates scalable on-demand expansion, and simplifies system upgrades and maintenance. In a quintessential System of Systems (SoS) environment like the LAE, where different elements are often developed and operated by various entities, a layered architecture supporting principles such as separation of concerns and abstraction is crucial. By defining standardized points of interaction, the architecture becomes a critical instrument for managing the lifecycle and continuous optimization of the LAE infrastructure.

### 2.2. Description of the Layered Framework: Proposed Multi-Layered Framework

**Figure 1:**
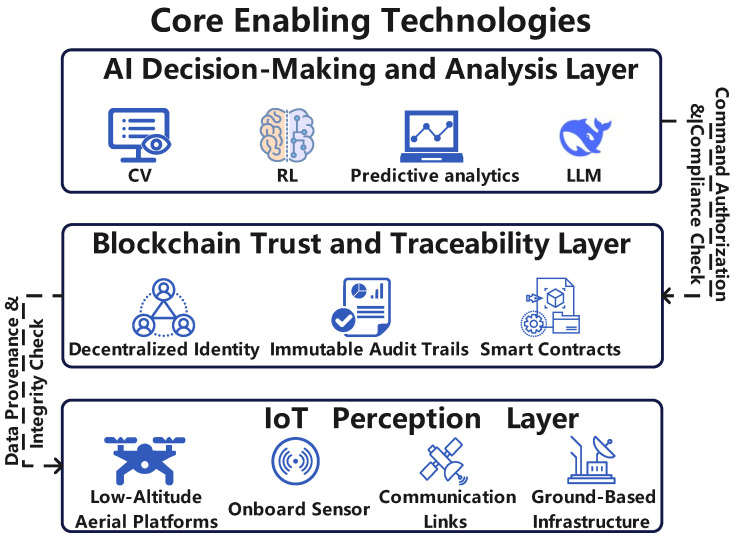
The IoT layer is the foundational layer, responsible for seeing and hearing, addressing the challenge of data provenance; the AI layer is responsible for processing thinking and decision-making, solving issues of data processing and intelligence; the blockchain layer manages recording and verifying, sitting between the IoT layer and the AI layer, ensuring the overall trustworthiness of system operations.

To address the multifaceted requirements of the LAE, this paper proposes a conceptual, multi-layered architectural framework, as depicted in Figure 1. This framework logically stratifies the functions necessary for secure and intelligent operations, promoting synergy between the IoT, AI, and blockchain. The architecture is divided into three primary layers: Physical and Perception Layer, Decision and Intelligence Layer, Trust and Service Layer.

Figure 1 illustrates the composition of the IoT, AI, and Blockchain layers, and the architecture for their synergistic integration.

#### 2.2.1. Physical and Perception Layer (The IoT Foundation)

This layer constitutes the fundamental interface with the physical world. It is responsible for comprehensive data acquisition from the operational environment and the execution of physical actions, serving as the perceptual and actuation backbone of the LAE. Its implementation is primarily based on the principles and technologies of the IoT.

Its core purpose is to generate a continuous, multi-modal stream of data describing the state of both the operational environment and the system’s physical assets. This includes, but is not limited to, aircraft telemetry, environmental conditions, airspace occupancy, and obstacle detection. Key components include diverse aerial platforms (e.g., UAVs [24], eVTOLs [3]), their onboard sensor suites (e.g., cameras [7,8], LiDAR [25,26]), extensive ground-based infrastructure (e.g., GCS [27], vertiports [28], weather sensors [29]), and a resilient communications network (e.g., 5G [30,31], satellite [32]). This layer provides comprehensive situational awareness to the upper layers and executes control instructions issued by the intelligence layer.

#### 2.2.2. Decision and Intelligence Layer (The AI Brain)

Positioned above the Physical and Perception Layer, the Decision and Intelligence Layer functions as the central processing and cognitive engine of the LAE infrastructure. Its core mission is to receive, process, fuse, and analyze the massive datasets transmitted from the layer below in order to make intelligent decisions, generate commands, and enable autonomous operations. In essence, it serves as the intelligence hub of the LAE.

Key capabilities, driven by AI models and algorithms, include the interpretation of complex sensor data for object recognition [33], the generation of optimized and conflict-free trajectories for autonomous navigation using techniques like Reinforcement Learning (RL) [34], predictive analytics for operational forecasting [35,36,37], and the detection of anomalous patterns indicative of safety or security risks [38,39,40]. This layer transforms raw data into actionable intelligence, thereby serving as the LAE’s intelligence hub.

#### 2.2.3. Trust and Service Layer (The Blockchain Ledger)

The Trust and Service Layer, which is founded on blockchain technology, provides trustworthy, secure, and transparent mechanisms for all participants, systems, and autonomous agents within the LAE ecosystem. It enables the automated execution of various services and agreements in a manner that is decentralized, verifiable, and immutable.

Core functions include providing cryptographically secure and self-sovereign digital identities (DIDs) for all entities [41], creating tamper-resistant, timestamped audit trails of critical operations for regulatory compliance and incident analysis [42], and automating multi-party agreements and transactions via smart contracts based on verified data inputs [22]. This layer replaces reliance on centralized authorities, fundamentally reshaping governance and value exchange within the ecosystem by embedding trust directly into the system’s architecture.

### 2.3. Data and Control Flow Dynamics

**Figure 2:**
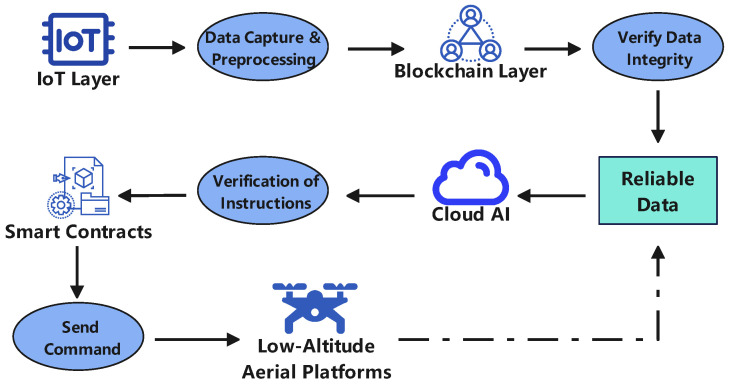
Functional Flow Diagram of the Integrated Infrastructure. This diagram illustrates the cyclical flow of data and control. (1) IoT devices capture and preprocess data at the edge. (2) Data integrity is verified by anchoring its hash on the blockchain. (3) Trusted data is fed to AI models executing on the edge or in the cloud. (4) AI-generated commands are validated against blockchain-based smart contracts. (5) Verified commands are sent to the UAV for execution, which generates new data and restarts the cycle.

The proposed layered architecture ensures the synergistic operation of the system through a dynamic, closed-loop flow of data and control, wherein the Trust and Service Layer plays a critical role of active verification and assurance at every stage. Figure 2 presents a detailed functional flow diagram that illustrates the operational synergy between IoT, AI, and Blockchain. This entire operational process can be summarized as an interactive cycle composed of Data Up-flow, Control Down-flow, and Feedback Loop.

In the Data Up-flow, heterogeneous data from the Physical and Perception Layer is captured by onboard sensors (e.g., cameras, LiDAR) and ground-based infrastructure. As shown in Step(1) of Figure 2, initial preprocessing, such as data filtering or compression, is often performed at the edge (i.e., on the UAV or at a ground station) to reduce latency and bandwidth consumption. Recognizing that this raw data originates from diverse sensors in varying formats, a crucial pre-processing and standardization step is required before it can be consumed by higher-level functions. Subsequently, before this standardized data is fed into AI models, the Trust and Service Layer intervenes (Step 2) to verify its provenance via DIDs and anchor its hash value onto the blockchain, establishing an immutable foundation for trustworthy decision-making. This trusted data is then processed by the Decision and Intelligence Layer (Step 3).

Subsequently, in the Control Down-flow, commands generated by the Decision and Intelligence Layer are sent to the Physical and Perception Layer for execution. Here, the Trust and Service Layer acts as a gatekeeper (Step 4). For instance, a smart contract can automatically verify if an AI-generated flight plan complies with regulations before the command is dispatched, ensuring the legitimacy of all control instructions.

Finally, the system adapts through a continuous Feedback Loop. The outcomes of executed commands (Step 5) are captured as new data, enabling AI models to learn from real-world performance. Concurrently, events from the Trust Layer, such as authentication failures, can trigger immediate responses at the decision or physical layers. This active, blockchain-based regulation embeds trust into every critical node, creating a resilient and secure operational environment.

## 3. Core Enabling Technologies

### 3.1. IoT Perception Layer

As established in the overall architecture, the IoT Perception Layer provides the empirical data foundation for the entire LAE system. This section now transitions from the conceptual framework to a detailed technical review of the core components that constitute this layer. We will examine the specific technologies that enable robust, real-time data acquisition, covering the primary hardware elements and communication links. The analysis will focus on diversified low-altitude aerial platforms, their advanced onboard sensor suites, and the essential ground-based infrastructure, illustrating how these elements interact to create a comprehensive perception network.

#### 3.1.1. Low-Altitude Aerial Platforms and Onboard Sensor Suites

Low-altitude aerial platforms function as mobile sensing nodes, with their onboard sensor suites acquiring high-precision, multi-dimensional information. The diversification of platform technologies and advancements in sensor technology have driven a significant leap in low-altitude perception capabilities, providing rich data sources for subsequent intelligent analysis and decision-making.

*Types of Low-Altitude Aerial Platforms.* The selection of a low-altitude aerial platform is not an isolated technical decision, but tightly coupled with specific mission requirements of the LAE and the types of sensors to be deployed. Different types of UAVs possess distinct flight characteristics, which in turn directly determine their suitability for various application scenarios. The main types of low-altitude aerial platforms include:

(1) **Multi-rotor UAVs:** This class of UAV is distinguished by its VTOL capabilities, stable hovering, high maneuverability and ease of operation, making such platforms exceptionally well-suited for executing close-range, high-precision tasks in complex or confined environments [43]. Typical applications include structural inspections of infrastructure such as high-rise buildings and bridges in urban environments [44], and continuous monitoring and data acquisition within localized areas [45,46]. In the domain of logistics, multi-rotor UAVs are frequently used for last-mile delivery [4]. However, their primary limitations are short flight endurance, typically ranging from 15 to 60 min, and low payload capacity. This limitation is further exacerbated by the significant power demands of advanced onboard sensor suites and the computational units required for real-time AI processing, making energy management a critical operational constraint. For instance, DJI’s Mavic 3 Pro UAV has a maximum flight time of approximately 43 min [47], constraining operational range and mission duration.
(2) **Fixed-wing UAVs:** Fixed-wing UAVs operate aerodynamic lift via their wings, offering high flight efficiency and long flight endurance. Some models, like the JOUAV CW-30E, can fly up to 480 min [48]. However, they typically require a runway or catapult for takeoff and either a glide path or parachute for recovery, imposing stricter site requirements. Additionally, they cannot hover and have poor low-speed maneuverability, necessitating more specialized pilot training. Typical application scenarios include large-scale geographic surveying and mapping [49], and the inspection of long-distance infrastructure, such as oil and gas pipelines [50].
(3) **VTOL:** VTOL UAVs merge the vertical takeoff and landing capabilities of multi-rotors with the long-endurance cruise of fixed-wing aircraft. This design eliminates runway dependence, significantly enhancing deployment flexibility while retaining extended endurance [51]. The unique performance advantages of VTOL UAVs make them highly promising for specific applications requiring both rapid response capabilities and significant area coverage, such as regional security surveillance, medium- to long-range logistics transport, and emergency response to unforeseen incidents [52,53]. Furthermore, the convergence of VTOL with the trends of electrification has spurred the development of eVTOL aircraft, specifically designed for UAM. These platforms, focusing on air taxi services and heavy cargo transport, represent a key frontier in LAE evolution and a significant step toward passenger-carrying applications [54].

*Onboard Sensor Suites.* The escalating demand for advanced environmental perception within the LAE is driving onboard sensor technology along two key dimensions. The first is Perceptual Depth, achieved by using higher-resolution sensors to capture granular visual information for morphological scene understanding and object recognition. The second is Data Dimensionality, which involves acquiring multi-dimensional information beyond human vision through sensors such as multispectral imagers, revealing the physicochemical properties and internal states of target objects. The fusion of diverse sensor types, tightly integrated with intelligent algorithms, is key to enhancing perception. The following are the commonly used onboard sensors for low-altitude platforms:

(1) **High-Resolution RGB Cameras:** High-resolution RGB cameras capture image data within the visible spectrum. Their resolution often exceeds 20 megapixels, for example, the DJI Mavic 3 UAV integrated a 4/3-inch, 20-megapixel sensor [47]. Their lightweight design extends drone range and endurance. In LAE applications, these cameras are widely used for infrastructure inspection (e.g., power lines, bridges, and buildings) to detect damage [7,8], monitoring crops growth status in precision agriculture [55], yield forecasting and weed detection [56,57,58,59], accident scene documentation [9], and target identification and tracking in security monitoring [60]. Photogrammetric techniques like Structure from Motion (SfM) allow sequential RGB images to generate high-precision 3D point clouds and Digital Elevation Models (DEMs) [61,62]. However, RGB cameras are limited by ambient lighting, weather, and poor penetration of dense vegetation. They also cannot directly acquire accurate 3D elevation data, often requiring complex post-processing.
(2) **LiDAR:** LiDAR is an active remote sensing technology that emits laser beams and measures the time difference between emission and return to calculate distances, generate high-precision 3D point cloud data. It offers centimeter-level 3D positioning accuracy and operates independently of ambient light, enabling both diurnal and nocturnal operations. Its laser beams can partially penetrate vegetation, with multiple return signals providing information on both canopy and ground surfaces [63]. LiDAR is widely applied in high-precision terrain mapping and 3D city modeling [25,64,65], power line safety analysis and vegetation intrusion detection [66,67], 3D scanning and deformation monitoring [68], obstacle detection and environmental perception in UAV navigation [26,69,70,71], and survey of forestry resources [72,73]. However, it faces challenges including high equipment cost, large raw data volumes, demanding UAV attitude control, and complex post-processing of point cloud data.
(3) **IR/Thermal Sensors:** IR/thermal sensors detect infrared radiation naturally emitted by objects and convert it into thermograms, visualizing surface temperature distributions. These sensors have extensive applications in LAE, particularly in Search and Rescue missions conducted at night or in low-visibility conditions [74,75]. In industrial inspections, they identify thermal anomalies in electrical systems to prevent failures [76], in wildfire prevention and management, detect incipient or concealed fire hotspots and track fire spread dynamics [77], in precision agriculture, they monitor crop canopy temperatures to assess soil moisture and drought stress [78,79]. However, thermal sensors typically offer lower spatial resolution than visible-light cameras, and their performance is influenced by ambient temperature and surface emissivity. Accurate data acquisition requires radiometric correction and careful parameter calibration.
(4) **Multispectral and Hyperspectral Sensors:** Multispectral and hyperspectral sensors capture an object’s reflected or emitted radiation across multiple narrow electromagnetic bands, offering far richer spectral data than RGB cameras. Multispectral sensors, with fewer bands and simpler processing, are widely used in practice. Hyperspectral sensors provide much higher spectral resolution, enabling subtle material identification and quantitative analysis, though they require complex algorithms, large storage, and high sensor costs. Both sensor types are central to precision agriculture for crop classification, growth and coverage assessment [56,80,81], crop quality assessment [82,83], pest and disease stress detection [84,85,86], and non-destructive nutrient monitoring [87,88]. In addition, they show potential in mineral resource exploration, environmental monitoring, and archeology [89,90,91]. The main limitation is that the amount of raw data is huge, and the data storage, transmission and processing capabilities are very high [92], and the demand of the accuracy of atmospheric radiative transfer correction is also high [93].
(5) **GNSS and IMU:** GNSS provides low-altitude aerial platforms with real-time geospatial position information and precise time references. The IMU measures platform attitude via internal gyroscopes and accelerometers. In practical applications, GNSS and IMU data are tightly coupled and fused through algorithms such as the Kalman filter to provide continuous and reliable navigation parameters, namely Position, Velocity, and Attitude (PVA) [94]. This fusion is essential for autonomous flight control and precise georeferencing of onboard sensor data. Consumer-grade GNSS typically offers meter-level accuracy, but Real-Time Kinematic (RTK) or Post-Processed Kinematic (PPK) techniques can enhance this to centimeter-level, vital for high-precision mapping and inspections [95]. IMU quality directly influences attitude solution accuracy and stability. Therefore, a high-precision GNSS/IMU system serves as the fundamental guarantee for both safe autonomous flight and the acquisition of accurately geolocated remote sensing data. Airborne LiDAR systems commonly integrate such modules to enable direct point cloud georeferencing with minimal or no ground control points [96,97].

Table 2 summarizes and compares the onboard sensor technologies commonly used for low-altitude platforms.

#### 3.1.2. Ground-Based Infrastructure, Sensor Networks and Communication Links

Ground-based infrastructure and sensor networks serve as the physical backbone for the safe and efficient operation of the LAE and act as a critical complement to its environmental perception capabilities. They not only provide essential services for the operation of aerial vehicles, such as command and control, takeoff and landing support, and energy replenishment, but also through the deployment of various ground-based sensors, work in synergy with aerial platforms to construct a more complete situational picture of the low-altitude environment. This collaboration enhances overall operational safety and efficiency. Particularly in complex urban environments, a robust ground infrastructure is a fundamental prerequisite for the realization of emerging business models such as UAM. The main components include:

(1) **Ground Control Stations:** The core hub for interaction between the UAV operator and system. Its primary functions include mission planning and route design, real-time flight monitoring, remote control command transmission, and data reception from the UAV [27]. A typical GCS comprises command and control software, communication hardware, computing and storage units, and an operator interface. Based on deployment configuration, GCS can be categorized as fixed or mobile. Fixed GCS are established in command centers for centralized fleet management. Mobile GCS, including vehicle-mounted and handheld variants, offer flexible, rapid deployment for dynamic mission requirements.
(2) **Vertiport Management Systems:** Vertiports are essential infrastructure within the Advanced Air Mobility (AAM) and UAM ecosystems, providing takeoff, landing, parking, passenger transit, cargo handling, and coordination with ATM and UTM systems [28]. A standard vertiport includes a Touchdown and Lift-off (TLOF) area, Final Approach and Takeoff (FATO) area, parking pads, charging facilities, a passenger terminal, cargo storage, and Maintenance, Repair, and Overhaul (MRO) areas. To enhance operational safety and efficiency, modern vertiport management systems integrate sensor networks and automation technologies. By deploying meteorological sensors and perimeter surveillance cameras, these systems perform real-time monitoring of the vertiport and its surroundings, guide aircraft approaches, optimize ground traffic, monitor charging status, and dynamically adjust operational schedules.
(3) **Environmental Sensing Ground Networks:** In addition to the onboard perception capabilities of aerial platforms themselves, specialized ground-based sensor networks are vital for ensuring the safety and efficiency of low-altitude flight. They provide environmental information that is broader in scope, more continuous, and more granular, thus complementing the data gathered by the aircraft.
  - **Weather Sensor Networks:** Low-altitude micrometeorology directly affects the flight safety, performance, and passenger comfort of small aerial vehicles. Traditional meteorological forecasts, limited in spatial and temporal resolution, cannot capture localized, rapidly changing micro-weather phenomena, making them insufficient for supporting UAM operations. To address this, a distributed sensor network comprising automated ground weather stations, Doppler LiDAR, and building-mounted micro-weather sensors should be deployed around vertiports and along flight corridors. These sensors enable real-time monitoring of key parameters such as wind speed, temperature, and humidity. By fusing multi-source observational data, fine-grained, real-time micro-weather information services can be generated. This data is essential for dynamic flight path planning and precise decision-making on takeoff and landing windows [29,98,99].
  - **Acoustic Sensor Networks:** The noise generated by low-altitude aircraft is a key environmental factor affecting public acceptance and social sustainability [100,101]. Continuous monitoring and management are therefore essential. By deploying acoustic sensor networks composed of high-precision microphones in sensitive areas such as residential zones, schools, and hospitals, a comprehensive regional noise monitoring system can be established [102]. These sensors record real-time noise spectral characteristics during overflights, enabling assessment of noise impact footprints, calibration of prediction models, and data-driven optimization of flight routes and operational strategies. This ensures compliance with environmental regulations and noise standards.
  - **Other Ground Sensors:** In addition to meteorological and acoustic sensors, ground-based infrastructure can integrate systems such as ground surveillance radar and electro-optical/IR cameras to detect, identify, and track unauthorized or anomalous aircraft, enhancing the security of the low-altitude environment [103].
(4) **Communication and Data Links:** A reliable communication network serves as the nervous system connecting ground-based infrastructure with aerial platforms. Since no single technology can meet the diverse demands of low-altitude applications, the integration of heterogeneous networks is essential. In densely populated areas, 5G and 5G-Advanced technologies, with low-latency and high-bandwidth, support high-density operations but face challenges such as insufficient low-altitude coverage and signal interference [30,31]. In remote regions, satellite communication is critical for Beyond Visual Line of Sight (BVLOS) flights [32], though it presents limitations in latency and terminal size. Future developments such as Integrated Sensing and Communication (ISAC) and integration with Non-Terrestrial Network (NTN) are expected to build a comprehensive space-air-ground intelligent connectivity network, enhancing system perception and coordination [104]. Thus, the LAE communication architecture must adopt a heterogeneously integrated system that dynamically combines multiple communication technologies based on mission needs and operational environments.

### 3.2. AI Decision-Making and Analysis Layer

AI is responsible for processing the massive, multi-modal data from the IoT layer to conduct analysis, decision-making, and autonomous control [105]. The deep integration of AI endows the entire low-altitude infrastructure with the capacity for intelligent and efficient task execution, comprehensively improving operational efficiency, precision, and safety [106]. Through advanced tools such as Machine Learning (ML) and Deep Learning (DL), AI equips low-altitude systems with powerful data processing and pattern recognition capabilities essential for managing complex, dynamic environments [107], ensuring flight safety [108], supporting precision agriculture [56,59,81], and enabling diverse commercial applications [109]. AI-driven systems further enhance operational safety through predictive maintenance (PdM), real-time anomaly detection, and automated emergency decision-making, mitigating risks inherent in autonomous operations. This section will examine the core AI branches applied in low-altitude infrastructure, focusing on how technologies such as CV, RL, predictive analytics, anomaly detection, and emerging LLMs collaboratively build a safe, efficient, and intelligent operational framework for the LAE.

#### 3.2.1. CV for Real-Time Perception

**Figure 3:**
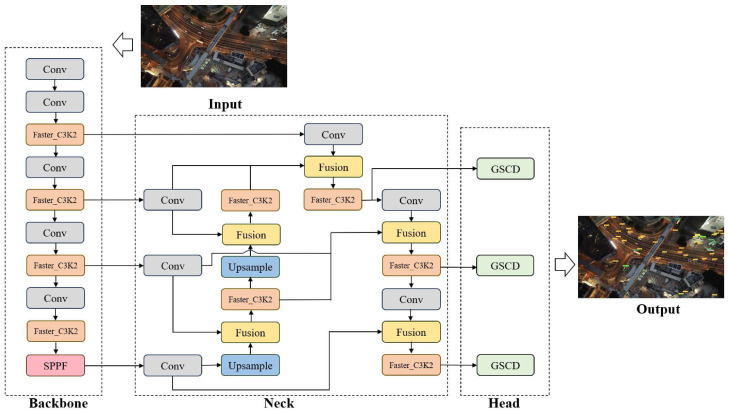
Framework of PS-YOLO [113].

**Figure 4:**
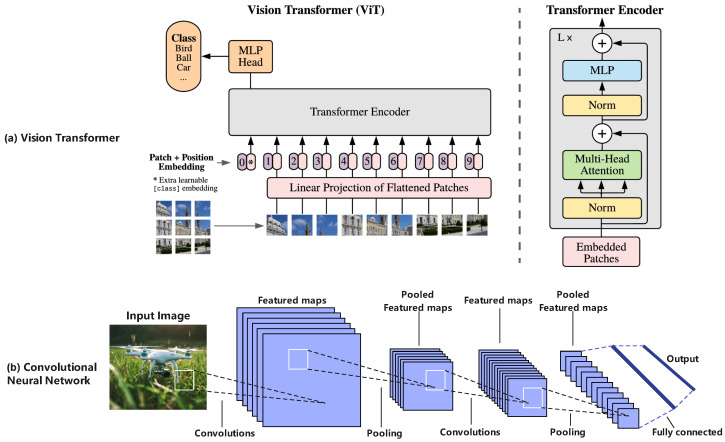
(**a**) ViT segments an image into a sequence of patches, capture long-range dependencies and global contextual information via self-attention mechanism [123]; (**b**) shows how CNNs rely on local convolutions.

CV serves as a core technology for environmental perception in low-altitude infrastructure. By performing real-time analysis of image and video data collected by aerial platform sensors, CV enables critical functions including object detection, tracking, autonomous navigation, infrastructure condition monitoring, and anomalous event identification [110,111,112]. However, CV in aerial imagery faces challenges like small object detection [113], cluttered backgrounds [114], scale variations [115], and motion blur [116]. To address these, researchers have developed various models:

(1) **CNN-based Models:** To address these challenges, researchers have developed various detection models based on Convolutional Neural Networks (CNNs). The YOLO (You Only Look Once) series of algorithms, renowned for its effective balance between speed and accuracy, is widely applied in real-time detection tasks [110,117,118,119,120,121,122]. Researchers have optimized YOLO models for UAV aerial imagery. For example, PS-YOLO [113] proposed a fast, accurate network for small object detection in UAV imagery. As shown in Figure 3, PS-YOLO employs several key innovations. It adopts a lightweight backbone network based on Partial Convolution (PConv), named Faster_C3k2, and utilizes a more efficient bidirectional feature fusion pyramid network, FasterBIFFPN. Additionally, it introduces a Gaussian Shared Convolutional Detection (GSCD) head and a Normalized Gaussian Wasserstein Distance Loss (NWDLoss) for bounding box regression.
(2) **ViT-based Models:** Vision Transformers (ViT) [123] and their hybrid variants are an emerging DL architecture increasingly applied to UAV image analysis and low-altitude perception. As shown in Figure 4, unlike CNNs which rely on local convolutions, ViT segments an image into patches and employs self-attention to capture long-range dependencies and global context. In UAV object tracking, ORTrack [124] maintains tracking stability under occlusion by learning Occlusion-Robust Representations (ORR). To address motion blur and resource constraints, BDTrack [125] introduces a Motion Blur Robust ViT (MBRV) with a Dynamic Early Exit Module (DEEM), which adjusts computation based on input complexity and improves feature extraction for blurred images. For object detection, ViTDet [126] utilizes a ViT as its backbone, demonstrating strong potential for aerial image analysis.
(3) **CNN-ViT:** To combine the strength of CNNs in extracting local details with the global modeling capabilities of ViT, BrownViTNet [127] proposes a hybrid CNN-ViT architecture, employs a CNN for shallow-feature extraction and a ViT for deep-level global relationship learning, achieving promising results in land use classification tasks for aerial imagery, such as brownfield identification.

For a clearer comparison of the progress among different CV models in the domain of low-altitude perception, Table 3 summarizes the key technical characteristics, advantages, limitations, application examples, and relevant references for major model categories.

The deployment of these CV models—whether directly on the UAV (onboard), at a nearby edge server, or in the cloud—is a critical design choice dictated by a trade-off between latency, model complexity, and communication reliability. For real-time, safety-critical tasks such as sense-and-avoid, lightweight models like optimized YOLO variants are often deployed onboard the UAV to minimize decision latency [133]. This approach ensures immediate response capabilities even in communication-denied environments. In contrast, more computationally intensive models, such as large ViTs used for complex scene analysis or post-mission data processing, are typically executed on ground-based edge or cloud servers where greater computational resources are available [134]. This hybrid approach, often termed cloud-edge-device collaborative inference, allows the system to balance the need for instantaneous reaction with the benefits of powerful, large-scale analysis, thereby optimizing both safety and operational intelligence.

#### 3.2.2. RL for Autonomous Path Planning and Control

RL is emerging as a key enabling technology for achieving advanced autonomous decision-making and intelligent control for unmanned systems within low-altitude infrastructure. Unlike traditional control methods, an RL agent learns through a trial-and-error mechanism. By continuously interacting with the dynamic low-altitude environment, it learns how to select actions that maximize a cumulative reward signal, thereby autonomously mastering the skills required to complete complex tasks such as autonomous navigation, dynamic path planning, and multi-agent coordination and confrontation.

(1) **Single Agent RL used for Path Planning and Flight Control:** In low-altitude applications, a single UAV can leverage RL techniques to perform autonomous path planning and flight control. The core objective is to plan an optimal trajectory and precisely control the aircraft’s flight along that path, subject to mission constraints such as time limits, energy consumption, and the avoidance of no-fly zones. Typical scenarios include autonomous navigation in urban canyons or unknown territories, real-time avoidance of static and dynamic obstacles, and dynamic trajectory optimization based on real-time energy consumption, battery levels, and mission priorities [135,136]. To achieve these functions, researchers explored a variety of RL algorithms and their applications in UAV path planning and control:
  - **Value-based Iterative Approach:** Deep Q-Network (DQN) and its extensions, such as Double DQN and Dueling DQN, use neural networks to approximate the state-action value function (Q-function), guiding the agent to select actions with the highest Q-value. These methods perform well for control problems with discrete action spaces and have been applied in simplified UAV path planning tasks [34].
  - **Strategy Gradient-based Approach:** Learning a parameterized policy function that maps states to actions or a probability distribution over actions. Actor-Critic algorithms enhance this by introducing a critic network to evaluate the policy, guiding updates to the actor network and reducing policy gradient variance. A representative example, the Asynchronous Advantage Actor-Critic (A3C), improves learning efficiency and stability by allowing multiple agents to train asynchronously in parallel across different environment instances. This method has been applied in UAV navigation tasks [137].
  - **Deep Deterministic Policy Gradient (DDPG):** A class of Actor-Critic algorithms tailored for continuous action spaces. It combines experience replay and target networks from DQN with deterministic policy gradients, enabling UAVs to learn precise, continuous control commands such as flight velocity, acceleration, and control surface deflection angles [138].
  - **Twin Delayed Deep Deterministic Policy Gradient (TD3):** A significant improvement upon DDPG, TD3 mitigates Q-value overestimation and training instability by introducing three techniques: Target Policy Smoothing, Clipped Double Q-Learning, and Delayed Policy Updates. It demonstrates superior performance and robustness in complex continuous control tasks for UAVs, such as local path planning and dynamic obstacle avoidance [139].
(2) **MARL for Collaborative Operations:** As low-altitude scenarios grow more complex, the capabilities of a single UAV are often insufficient. UAV swarms, through cooperative coordination, can tackle tasks beyond the capacity of individual agents [140]. MARL offers a robust framework for autonomous swarm coordination, where each UAV operates as an independent agent. By interacting with the environment and peers, agents learn to autonomously adjust their policies to collectively optimize global or local objectives. Typical applications for MARL in low-altitude infrastructure are wide-ranging, including:
  - **Regional Coverage and Collaborative Exploration:** In tasks such as post-disaster SAR, a UAV swarm can leverage MARL to learn efficient, collaborative exploration strategies. The objective is to cover an unknown area with maximum speed and minimal redundancy, ensuring no critical information is missed. For example, in an earthquake zone, a swarm could autonomously partition the area into sub-regions and share real-time information on detected signs of life or damage, greatly improving SAR efficiency [141].
  - **Dynamic Target Collaborative Tracking and Monitoring:** In scenarios involving highly mobile targets, a UAV swarm can leverage MARL to learn collaborative tracking strategies. This enables the swarm to dynamically adjust positions and formations to maintain continuous, stable surveillance of the target [142].
  - **Coordinated Transportation and Material Delivery:** For heavy or oversized items that a single UAV cannot transport, UAV swarm can perform a cooperative lift and transport mission. MARL enables them to coordinate thrust outputs and flight trajectories in real time, ensuring payload stability and formation integrity throughout the transport process, and allowing safe delivery to the designated location [143].
  - **Precision Agriculture and Environmental Monitoring:** In large-scale agricultural fields or complex ecological environments, UAV swarms can conduct collaborative operations. MARL optimizes flight paths and task allocation to avoid redundancy and improve overall monitoring efficiency [144].
  - **Communication Relay and Self-organizing Network Construction:** In areas where terrestrial communication infrastructure is damaged or coverage is insufficient, UAVs can serve as aerial mobile base stations or relay nodes to establish a temporary wireless network. MARL dynamically optimizes UAV deployment positions, connection topology, and wireless resource allocation to provide reliable communication services for ground users [145].

The execution environment for RL and MARL algorithms is mission-dependent and aligns with a hierarchical control strategy. For a single agent, high-level mission planning and global trajectory optimization can be performed pre-flight in the cloud, leveraging extensive computational power and data. However, for dynamic obstacle avoidance and real-time flight control adjustments, the RL inference loop must run with minimal latency, favoring deployment on powerful onboard processors or proximate edge servers. In MARL-based swarm operations, a decentralized execution model is common, where each UAV runs its own policy onboard while communicating with peers via Vehicle-to-Everything (V2X) links to achieve coordination. Alternatively, a centralized or hybrid model can be employed, where a ground-based edge server acts as a central coordinator, collecting state information from the swarm and broadcasting commands. This architectural choice is determined by the required level of coordination, communication constraints, and the complexity of the collective task.

Table 4 summarizes the main types of RL algorithms used for UAV path planning and swarm coordination and their characteristics.

#### 3.2.3. Predictive Analytics and Anomaly Detection to Enhance Security and Efficiency

In the complex operational environment of low-altitude infrastructure, leveraging ML techniques for predictive analytics and real-time anomaly detection is strategically vital. These capabilities ensure flight safety, enhance operational efficiency, maintain system integrity, and support intelligent management. By analyzing historical and real-time data from sensor networks, aerial vehicles, and external systems, ML can issue early warnings of potential risks, detect behavioral anomalies, and deliver data-driven insights to planners, managers, and operators of the low-altitude system.

(1) **Predictive Analytics:** Leverages historical data and statistical models to forecast future events and trends, enables the managers of low-altitude infrastructure to shift from a passive, event-response model to a proactive, risk-mitigation paradigm. ML techniques play a vital role in predictive analytics for low-altitude infrastructure operations. By analyzing historical flight data, meteorological records, and special events, ML models can forecast future air traffic flow, airspace congestion levels, and potential flight conflicts [35]. Integrating historical and real-time meteorological data with past incident records also enables the prediction of adverse weather phenomena that threaten flight safety, allowing systems to issue timely warnings, adjust flight plans, and select alternate airports as needed [36]. Additionally, by monitoring sensor data from infrastructure equipment alongside operational and maintenance histories, ML can predict a component’s Remaining Useful Life (RUL), failure probabilities, and performance degradation trends [37]. This PdM approach allows proactive scheduling of repairs and replacements, reducing costs, extending equipment lifespan, and improving system reliability.
(2) **Anomaly Detection:** The process of identifying data points within massive datasets that deviate significantly from normal patterns or expected regularities. In the context of low-altitude infrastructure, anomaly detection acts as a “firewall”, enabling the timely identification of potential threats and failures to maintain operational safety and order. By analyzing real-time and historical UAV flight trajectory data, DL models can identify abnormal flight patterns that deviate from typical behaviors, enabling timely alerts to regulatory authorities [38]. Additionally, by continuously monitoring sensor data from UAVs and ground infrastructure, anomaly detection algorithms can detect early signs of system faults or impending component failures [40]. In parallel, analyzing network traffic, connection behaviors, protocol interactions, and system logs allows for the identification of cybersecurity threats, including unauthorized access, data breaches, and malicious attacks, safeguarding the integrity of low-altitude communication networks [39].

#### 3.2.4. Emerging Role of LLMs in Intelligent Operations

Leveraging their exceptional capabilities in natural language understanding, text generation, knowledge question-answering, logical reasoning, and in-context learning, LLMs demonstrate immense application potential for the intelligent operation and management of low-altitude infrastructure. LLMs are poised to empower the autonomous planning and execution of advanced, complex tasks and assist operators in conducting high-quality analysis and decision-making by providing more natural and intuitive methods of human–machine interaction. This, in turn, is expected to significantly enhance the intelligence level, operational efficiency, and user experience of the entire low-altitude system.

*Main Applications of LLMs in Low-altitude Infrastructure.* LLMs play a crucial role in enhancing high-level mission planning, human–machine interaction, and decision-making within low-altitude systems. Operators can issue natural language commands, which LLMs translate into executable UAV task code, as demonstrated by frameworks like FLUC [152] and LLM-QTRAN [151], enabling both individual UAV control and dynamic swarm task allocation. Additionally, LLMs facilitate advanced human–machine interaction by processing operator queries, integrating multi-source data, and delivering comprehensible responses, such as in the Neuro-LIFT framework [153], which converts voice commands into real-time UAV flight instructions. Furthermore, through integration with techniques like Retrieval-Augmented Generation (RAG), LLMs can access and incorporate domain-specific knowledge—covering aviation regulations, maintenance protocols, and emergency procedures—into task execution and decision-making, thereby providing expert-level operational support [154].

Table 5 summarizes some emerging application, role, advantages, challenges, and related research progress of LLM in low-altitude infrastructure management.

*Challenges and Operational Constraints for LLM Deployment.* Despite their transformative potential, the direct application of LLMs in low-altitude infrastructure faces significant real-world constraints that temper expectations and define clear areas for future research. These challenges stem from the inherent characteristics of current-generation LLMs and the stringent demands of aviation environments. State-of-the-art large language models (LLMs) contain billions of parameters and demand immense computational resources, far exceeding the capabilities of resource-constrained UAV platforms [157]. Consequently, inference is typically offloaded to cloud or ground-based servers, introducing considerable round-trip latency that encompasses data transmission, remote processing, and feedback delivery [158]. Such delays are unacceptable for safety-critical, real-time operations like dynamic collision avoidance or emergency maneuvers, where sub-second responses are imperative [159]. Moreover, cloud dependency requires persistent, high-bandwidth, low-latency connectivity—conditions that are often compromised in remote regions, dense urban areas, or environments affected by electromagnetic interference. Disruptions in air-to-ground communication can therefore sever a UAV’s link to its cognitive core, resulting in mission degradation or potential safety risks [159]. Finally, the direct deployment or “onboarding” of LLMs onto UAV flight controllers remains infeasible under strict power, weight, and thermal constraints. Although emerging lightweight models offer partial solutions, they generally lack the comprehensive reasoning and adaptability of their larger counterparts, highlighting the unresolved trade-off between onboard autonomy and computational performance.

*Deep Transformative Potential of LLMs in low-altitude Intelligent Operations.* The advent of LLM represents more than just an additional AI tool for low-altitude infrastructure; it holds the potential to fundamentally reshape system design philosophies, operational models, and human-system relationships, warranting forward-looking and strategic consideration.

Firstly, LLMs are poised to become the core natural language interface and intelligent task orchestration hub for future low-altitude systems. At present, operating such systems requires specialized domain knowledge and interaction via complex Graphical User Interfaces (GUIs), command-line interfaces, or programming APIs, resulting in high barriers to entry and limited efficiency [160]. In contrast, LLMs can translate user intent and constraints expressed in natural language into machine-executable instructions [152,153]. With the advancement of multi-modal understanding, reasoning, tool use, and agent collaboration, LLMs will evolve beyond “language-to-code” translation into a “master task orchestrator” connecting and scheduling heterogeneous infrastructure components [155]. In this paradigm, users describe their requirements via natural language, and the LLM autonomously interprets intent, invoking functional modules like object detection or path planning for efficient, safe mission execution [152]. This would lower operational complexity, enable non-specialists to deploy missions, and broaden the scope of LAE applications, establishing LLMs as the interaction and control core of future low-altitude intelligent ecosystems.

Secondly, integrating LLMs will reshape the “human–machine-environment” relationship within low-altitude infrastructure. Traditional systems rely on human-dominated, machine-passive workflows treating the environment as a reactive object. LLMs enable machines to proactively engage in human–machine interaction and task execution [153], integrating multi-source information to better understand environmental dynamics [161]. This bidirectional awareness allows LLM-driven systems to autonomously plan actions and, when necessary, engage in collaborative decision-making with human operators [152]. This fosters a new paradigm where humans define high-level objectives while LLM agents autonomously execute tasks and collaborate with operators in complex scenarios, enhancing mission autonomy, flexibility, and environmental adaptability.

### 3.3. Blockchain Trust and Traceability Layer

In the increasingly complex and dynamic ecosystem of the LAE, establishing a mechanism for trust and traceability is of critical importance for ensuring operational safety and fostering multi-party collaboration. Blockchain technology, as a decentralized, immutable, and transparent form of Distributed Ledger Technology (DLT), provides the core technical underpinning for building this crucial mechanism. It employs cryptographic methods to guarantee data integrity and the reliability of its provenance, making it possible to establish trust in a distributed network environment that lacks a central authority. However, the choice of underlying blockchain technology, particularly its consensus mechanism, must be carefully evaluated to meet the stringent real-time performance and energy constraints inherent in low-altitude operations. Energy-intensive mechanisms like Proof-of-Work (PoW), common in public blockchains like Bitcoin, are unsuitable for LAE infrastructure due to their immense computational power requirements [162]. Deploying such protocols would unacceptably drain the limited battery resources of UAVs and other mobile IoT devices. Consequently, the LAE context necessitates the adoption of lightweight and energy-efficient consensus algorithms, such as Proof-of-Authority (PoA), Delegated Proof-of-Stake (DPoS), or other Byzantine Fault Tolerance (BFT) variants, which are better suited for permissioned or consortium chains. This represents a critical design trade-off where the decentralization model is tailored to prioritize operational viability and energy efficiency over the complete trustlessness offered by PoW. This section will focus on elucidating how blockchain provides a solid foundation of trust for low-altitude infrastructure through its core components: DID, Immutable Audit Trails, and the automation capabilities of Smart Contracts.

#### 3.3.1. Decentralized Identity

DID is an emerging digital identity paradigm that provides unique, self-sovereign, and cryptographically verifiable identifiers for entities within low-altitude infrastructure. Its decentralized nature shifts identity management control from centralized authorities to identity subjects, enhancing system resilience and tamper-resistance [41]. This prevents identity spoofing and unauthorized access. For example, a UAV can use its DID to prove legitimate registration and compliance, while an operator securely presents operational credentials without relying entirely on a vulnerable central server [163].

From a technical implementation perspective, a DID typically manifests as a Uniform Resource Identifier (URI) composed of a DID method and a method-specific identifier. It can be resolved by a DID resolver into a “DID document” recording associated public keys, verification methods, and service endpoints, which is stored on a Verifiable Data Registry (VDR). Currently, several mainstream implementation paths exist. The blockchain-based Sidetree protocol enhances scalability and immutability by batching multiple DID operations and anchoring them to a blockchain, making it suitable for large-scale UAV identity registration and frequent updates. Permissioned chain DID methods operate in consortium or private chain environments, granting authorized organizations CRUD permissions to meet regulatory or enterprise compliance requirements. DID:WEB leverages existing DNS and web infrastructure for easier deployment and adoption. Peer DID enables peer-to-peer exchange of DID documents without a central registry, ideal for privacy-sensitive scenarios like secure UAV-operator communication.

When deploying a DID system within low-altitude infrastructure, multiple DID methods and trust models are expected to coexist, as no single solution can meet all requirements. Different entities have varying identity needs across scenarios. For example, a UAV performing critical infrastructure inspection is suited to a permissioned chain DID with strong governance [164], while wide-area, low-cost environmental sensors may adopt lightweight DIDs managed by IoT gateways [165]. This heterogeneity necessitates prioritizing interoperability standards among different DID methods to avoid identity silos and maintain the value of a unified identity infrastructure. Promoting DID standardization and developing cross-chain interoperability protocols are essential to ensure seamless identity verification within the LAE ecosystem. Simultaneously, DID security requires dynamic maintenance, with comprehensive lifecycle management crucial for sustaining trust. Given the likelihood of credential leakage and frequent status changes in low-altitude environments, a robust DID system must support credential issuance, updates, revocation, and recovery [166]. Without effective management, identity abuse could threaten airspace security. Maintaining a real-time, authoritative registry of trusted organizations and identity statuses is vital for preserving data validity and privacy protection [167].

#### 3.3.2. Immutable Audit Trails

Through its distributed consensus mechanism and chained-block structure, blockchain technology ensures that once data is recorded on-chain, it is extremely difficult for any entity to tamper with or delete it. This inherent immutability provides a credible and traceable audit log for critical activities and data changes within low-altitude infrastructure. Such capability is vital for meeting stringent regulatory requirements. In the event of a safety incident, violation, or dispute, a blockchain-based audit trail offers clear, chronologically ordered, and cryptographically verifiable records, simplifying incident investigation and liability determination [168].

However, merely recording data on-chain does not guarantee the reliability of an audit trail. Although blockchain ensures post-record immutability, it cannot verify data authenticity prior to commitment, introducing the “Garbage In, Garbage Out” problem. Certification schemes, such as that proposed by Li et al., emphasize establishing data quality gatekeepers before on-chain recording [169]. This involves technical measures like sensor data fusion, anomaly detection at edge nodes [170], and procedural mechanisms such as multi-party digital signatures [171]. Without pre-validation, immutable on-chain errors could mislead regulatory reviews. Integrating blockchain audit trails with IoT-level quality control and AI pre-processing is essential for ensuring data integrity and driving the development of trusted sensors, multi-signature edge nodes, and relevant standards. Moreover, immutable records of flight, sensor, and maintenance data provide high-quality inputs for AI-based UAV health assessments and PdM [169]. On-chain logs enable early failure detection, RUL prediction, and optimized maintenance [172], making blockchain not only a tool for traceability but also a foundation for proactive safety management. Alabadi et al. [173] exemplified this by combining DL, blockchain, and distributed storage for dynamic RUL prediction with ensured data security.

Finally, audit trail systems must balance data privacy with transparent auditing. In low-altitude operations, data like flight paths and cargo manifests are highly sensitive. Public on-chain disclosure risks privacy breaches, discouraging data sharing. Solutions include PrivChain’s Zero-Knowledge Range Proofs (ZKRP) [174], zk-SNARKs for encrypted UAV compliance audits [175], and smart contract-based dynamic Role-Based Access Control (RBAC) [176]. Shafagh et al. [177] proposed off-chain log storage with on-chain cryptographic proofs and encrypted data streams. Overall, a hybrid architecture combining on-chain governance with off-chain data management, preferably on a consortium or permissioned chain, achieves a dynamic privacy–transparency balance, enhancing system trustworthiness and promoting adoption.

#### 3.3.3. Smart Contracts for Automation

Smart contracts are self-executing segments of code deployed on a blockchain, which automatically enforce agreements based on preset rules without human intervention. In the context of low-altitude infrastructure, they significantly enhance process automation, reduce operational costs, and improve trust and collaborative efficiency among participants. By embedding agreements in code, smart contracts offer an efficient and reliable solution for complex interaction scenarios within the LAE. Typical applications include automated airspace leasing, dynamic geofencing compliance checks, payment-upon-delivery, and automated flight insurance claims.

The effective execution of smart contracts depends on accurate inputs from the physical world, typically provided by “oracles”. To ensure data privacy and authenticity, the zk-AuthFeed framework [178] employs zk-DASNARK technology to establish a zero-knowledge authenticated data feed, enabling offline computation with on-chain verification, while digital signatures guarantee data integrity. In low-altitude infrastructure, IoT device networks act as primary data sources for oracles. Smart contracts can also reliably execute AI-generated decisions, as demonstrated in blockchain-based UTM systems, where contracts automatically implement commands for airspace optimization or flight path adjustments. Integrating mechanisms like Mobile Crowdsensing (MCS) further enhances automation and airspace security [22].

Beyond process optimization, the automation and trustless nature of smart contracts enable entirely new business models for the LAE. For example, a P2P airspace sharing platform could allow idle airspace resources to be temporarily leased to UAV operators, with leasing, authorization, and billing handled automatically [22,179]. Similarly, on-demand sensor data markets based on micropayments could allow UAVs to purchase fine-grained environmental data from nearby drones or ground sensors in real time to optimize mission execution [180]. These models improve resource utilization, lower entry barriers, and stimulate innovation. In such scenarios, smart contracts act as trusted, automated engines for transaction matching, value measurement, and proceeds distribution, making small-value, high-frequency services feasible. This positions blockchain not merely as a technical support layer but as a catalyst for business model innovation and ecosystem evolution, expanding the boundaries and value of the LAE.

### 3.4. Summary

This section outlines the foundational technologies supporting secure and intelligent low-altitude infrastructure, centered on three layers: IoT perception, AI decision-making, and blockchain-based trust. The IoT layer integrates aerial platforms, advanced onboard sensors, and ground-based infrastructure to build a real-time, multi-source sensing network. The AI layer applies techniques such as computer vision, reinforcement learning, and predictive analytics to enable autonomous navigation, anomaly detection, and system optimization. Emerging large language models (LLMs) further enhance human–machine interaction and high-level task planning. Finally, the blockchain layer ensures trust and traceability through decentralized identity, audit trails, and smart contracts, while enabling automation and new business models. Together, these layers form a cohesive framework for intelligent and resilient LAE systems.

## 4. Typical Application Scenarios

This section aims to explore the practical applications of an integrated infrastructure, a trinity of the IoT, AI, and blockchain technologies, within the LAE. By analyzing the core challenges across different scenarios, this section will elaborate on the specific role of each technology within this synergistic framework, synthesizing findings from the latest academic research and industry projects. The analysis indicates that this technological triad not only possesses significant theoretical potential but also provides robust solutions for addressing the complex, real-world problems of the emerging LAE.

To clearly demonstrate the synergy of various technologies in different scenarios, Table 6 summarizes the application scenarios that will be discussed in this section.

### 4.1. Urban Logistics and Instant Delivery

#### 4.1.1. Problem Statement: The Challenging Last-Mile Delivery and the Rise of the On-Demand Economy

In the supply chain, “last-mile” delivery is traditionally the most costly and inefficient segment. With the rise of e-commerce and instant delivery services, coupled with rising labor costs and urban traffic congestion, has placed unprecedented pressure on conventional logistics systems [187]. Poorly managed parcel volume leads to increased operational costs, higher carbon emissions, and a decline in quality of life, with failed deliveries and low automation being key contributing factors [187,188]. At the same time, policymakers generally lack effective tools for real-time visualization and management of delivery networks [187]. Particularly in on-demand hyperlocal market, which remains a lack of systematic exploration into UAVs can be leveraged for sustainable and efficient operations [189]. Therefore, there is an urgent need to construct a novel technological infrastructure to address these complex challenges.

#### 4.1.2. Integrated Solutions: An Autonomous and Verifiable Distribution Network

**Figure 5:**
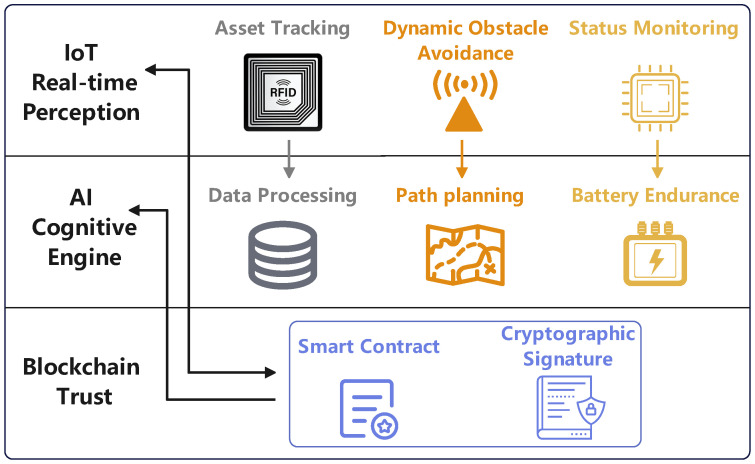
By synergizing IoT, AI, and blockchain, a highly automated, self-optimizing, and fully trusted distribution network can be constructed, transforming logistics from a series of disjointed steps into a seamless, verifiable, and intelligent process.

As shown in Figure 5, an integrated infrastructure that works together with IoT, AI, and blockchain enables the construction of a highly automated, self-optimizing and fully trusted urban logistics network.

*IoT as Real-time Perception Layer.* IoT serving as the key bridge between the physical and digital worlds, provides low-altitude infrastructure with real-time, high-granularity perception capabilities that extend far beyond GPS, forms the data foundation for both AI decision-making and blockchain-based trust mechanisms [190,191]. The construction of an IoT perception layer within this infrastructure marks a paradigm shift from isolated data points to a real-time, integrated ecosystem. The system is no longer merely collecting data, it is constructing a digital twin of the low-altitude environment and its associated assets. This comprehensive digital representation is a prerequisite for AI-driven decision-making, enabling a transition from passive, reactive operational modes to a model of proactive optimization.

In the domain of asset and environmental tracking, low-altitude systems utilize GPS and Radio-Frequency Identification (RFID) sensors deployed on UAVs and packages to achieve real-time asset location tracking. As the number of UAVs increases, the demand for enhanced situational awareness is driving the evolution of tracking systems from basic GPS to more reliable solutions, reflecting a rise in operational complexity and safety requirements [192]. RFID technology has expanded its functionality to include integrated sensing. For instance, chipless RFID humidity sensors embedded within smart packaging can wirelessly monitor a package’s internal humidity to ensure cargo integrity, a capability that transcends simple location tracking [193]. This demonstrates that the IoT perception layer not only tracks the aircraft itself but also monitors the condition of its payload, providing critical support for logistics and temperature-controlled transport. Simultaneously, onboard meteorological sensors continuously collect data such as temperature and humidity along the flight path. Through high-resolution environmental sensing, these sensors can identify weather hazards like freezing precipitation, thereby enhancing both flight safety and route planning capabilities [194,195].

For dynamic obstacle avoidance, UAVs are equipped with high-resolution cameras and LiDAR to perform real-time detection of dynamic obstacles within their flight path. LiDAR provides precise 3D geometry and depth information, which is supplemented by the semantic context from the cameras. The fusion of these two data streams enhances detection accuracy and environmental robustness, providing critical input for AI decision-making [196]. This exemplifies the key trend of shifting towards multi-modal sensor integration to achieve superior environmental perception.

In terms of status monitoring and communication, internal sensors continuously monitor a UAV’s key health parameters to support PdM and provide early fault warnings [194]. For instance, dynamically monitoring liquid levels during a spraying operation ensures both operational continuity and equipment safety [197]. Simultaneously, V2X communication modules enable UAVs to interact in real time with infrastructure, such as traffic lights, and with other vehicles [198]. Based on protocols like 5G and ITS-G5, this capability enhances situational awareness and autonomous coordination [199].

In summary, the comprehensive integration of a diversified IoT sensor network constitutes the cornerstone of a safe and intelligent low-altitude infrastructure. By providing real-time, high-granularity data across all operational levels, the IoT acts as the “eyes and ears” of the LAE. This continuous, multi-modal data stream is the essential prerequisite for both AI-driven decision-making and blockchain-based trust mechanisms, ensuring safe, efficient, and scalable operations within complex urban and rural low-altitude environments [200].

*AI as Cognitive and Optimization Engine.* AI effectively addresses the challenges of routing and resource management in urban logistics by transforming the massive data collected from IoT sensor networks into optimized decisions. AI models have evolved from static, single-factor optimization to dynamic, multi-factor decision-making [201]. In the domain of path optimization, AI algorithms have expanded from the basic Vehicle Routing Problem (VRP) to encompass the Traveling Salesman Problem (TSP) with Drone and collaborative truck-and-drone delivery systems [202,203]. These systems treat trucks as mobile aerial “motherships” and enhance delivery efficiency through the synchronized optimization of ground and air transport capabilities [204,205]. Modern AI models can incorporate complex variables such as time-dependent routes, stochastic demand, no-fly zones, and variable speeds into their decision-making calculus [206]. Furthermore, Mixed-Integer Linear Programming (MILP) is widely applied to path planning and energy consumption optimization, with the goal of minimizing travel distance and improving delivery efficiency [207].

AI optimization capabilities have extended to the management of critical resources. With battery endurance being a key bottleneck for UAV operations, AI models now integrate decisions such as battery charging and swapping directly into the routing optimization problem [208]. The system can monitor battery status in real time and dynamically schedule charging or replacement to achieve optimal global resource allocation [209]. Bhuiyan et al. demonstrates that intelligent battery management systems significantly reduce downtime caused by endurance limitations, thereby enhancing operational continuity and efficiency [210]. This type of integrated resource management strategy has become a key enabler for achieving large-scale and sustainable logistics operations.

At a more macroscopic level, AI in logistics is being integrated into pioneering frameworks such as the Physical Internet (PI). This concept envisions logistics networks as open, interconnected systems where AI serves as the central coordinator for various components, including urban logistics, last-mile delivery, and vehicle routing [211]. The integration of blockchain technology further enhances this framework by improving the system’s transparency, security, and traceability [20]. The PI model significantly reduces global logistics costs, improves infrastructure utilization rates, and enhances the system’s resilience to unforeseen events [212]. This innovative logistics paradigm is guiding the industry’s transition from siloed operations to a collaborative, shared ecosystem, thereby contributing to the development of future intelligent and sustainable urban logistics systems.

Furthermore, through the deep integration of IoT sensor networks with AI decision-making systems, modern logistics has achieved unprecedented levels of intelligence and efficiency. This synergy not only optimizes delivery routes and resource scheduling but also gives rise to novel collaborative delivery models and system architectures [213,214]. Looking ahead, AI-driven logistics optimization is set to evolve in a more dynamic, adaptive, and sustainable direction, providing robust support for the challenges of complex urban logistics.

*Blockchain as Immutable Layer of Trust and Transactions.* By virtue of its decentralized, cryptographically secure, and immutable characteristics, blockchain technology builds a solid foundation of trust for highly automated intelligent systems, addressing the trust deficit inherent in autonomous operations [215]. In low-altitude infrastructure applications, such as UAV delivery, blockchain enables a seamless link between delivery verification and automated settlement via smart contracts. For instance, when an IoT sensor writes successful delivery data to the blockchain, a smart contract can automatically trigger the payment process, releasing funds to the merchant. This creates a trusted, closed-loop transaction that requires no third-party intervention [216]. This blockchain-based automated payment mechanism significantly improves transaction efficiency while reducing both intermediary costs and the risk of potential human error or interference.

From the perspective of data integrity and end-to-end accountability, blockchain technology enables every critical node in the logistics process, from order generation and UAV dispatch to flight trajectory and final delivery, to be immutably recorded on a distributed ledger. This end-to-end, tamper-resistant audit trail is of significant value for resolving potential disputes and processing insurance claims [217,218]. Research by Kumar et al. [219] demonstrates that a blockchain-based supply chain tracking system can ensure the complete path of a product, from production to delivery, is securely and transparently recorded and verified. Each step is logged as an unalterable block, providing a technical guarantee for full-chain traceability.

In terms of secure communication and order authenticity, blockchain frameworks utilize cryptographic signature technology to safeguard the security of communications among all participants [220]. The integrity of order information and delivery instructions is verified through digital signatures, effectively preventing malicious attacks such as data tampering. In UAV delivery scenarios, the combination of blockchain and IoT sensors not only enhances system security but also increases the transparency and credibility of the delivery process [221,222]. The integrated application of blockchain and IoT has already expanded into multiple domains, such as smart cities and healthcare, providing technical support for data integrity and secure transactions [223].

In summary, blockchain, acting as an immutable trust and transaction layer, enables delivery verification and automated settlement through smart contracts, ensures data integrity and end-to-end accountability through its distributed ledger, safeguards secure communication and order authenticity through cryptographic signatures.

The logical chain of this technological fusion is clear: the IoT collects the facts, AI makes optimal decisions based on those facts, and blockchain ensures the authenticity and non-repudiation of both the facts and the decisions. If IoT sensor data is maliciously tampered with, the AI may make erroneous decisions. Thus, the role of blockchain must evolve from merely verifying the final result to continuously validating the integrity of the input for AI decisions. By enabling trusted IoT devices to write their data directly to an immutable ledger, the system can ensure that the AI operates on the basis of a tamper-resistant digital reality. Therefore, in a highly optimized, AI-driven system, blockchain is not only a trust layer that facilitates transactions, but also the foundational security layer that guarantees the integrity of the entire intelligent operation.

### 4.2. UAM and Intelligent Surveillance

With technological advancements, low-altitude airspace is beginning to be utilized for passenger transport and large-scale urban surveillance. This section will explore how an integrated infrastructure can ensure safe, scalable, and trustworthy operations within these high-risk, high-density application scenarios.

#### 4.2.1. Problem Statement: Crowded, Competitive, and High-Risk Airspace

UAM operating through a network of aerial vehicles and drones, offers new mobility and surveillance solutions for cities. It is widely regarded as an important means of alleviating ground congestion and promoting green transportation. However, its large-scale commercialization confronts complex obstacles, including challenges in infrastructure development and in energy and battery technology [224]. UAM systems are highly dependent on IoT sensors and communication networks, making them vulnerable to cyberattacks. Malicious tactics like data tampering and GPS spoofing could lead to a loss of aircraft control. As Wei et al. [225] point out, UAV systems possess multiple security vulnerabilities in their communication and navigation components, which malicious actors could exploit to cause severe consequences. Furthermore, the sensitive visual data generated by the deployment of smart surveillance within UAM systems raises public concerns regarding visual pollution and potential privacy breaches [226].

To address these challenges, researchers are developing a new generation of ATM systems that integrate AI, edge computing, and blockchain technology to enable the safe and efficient management of low-altitude airspace.

#### 4.2.2. Integrated Solutions: Build a Resilient and Trusted ATM System

**Figure 6:**
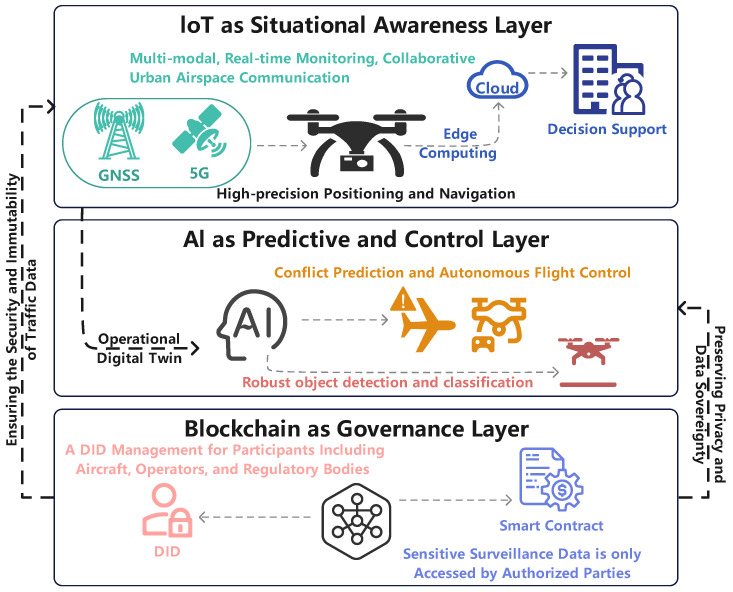
Implementation process for UAM and intelligent surveillance.

To address the challenges above, a comprehensive ATM system is required, one that is capable of enabling situational awareness, intelligent decision-making, and trustworthy governance. The process is shown in Figure 6.

*IoT as All-encompassing Situational Awareness Layer.* The IoT network, through the deployment of diverse sensor types, establishes a comprehensive, real-time situational awareness capability for urban low-altitude airspace, serving as the “eyes and ears” for the safe operation of UAM systems [227].

In the domain of high-precision positioning and communication, research by Wang et al. demonstrates that in urban settings, the fusion of GNSS with 5G-assisted positioning can reduce 3D positioning errors to the sub-meter level. This significantly enhances positioning accuracy and robustness, mitigating the problem of satellite signal obstruction [228]. Regarding intelligent surveillance sensor networks, UAV platforms integrate high-resolution optical, thermal, and multispectral cameras to support the multi-modal monitoring of traffic, crowds, and the environment. Data from these sensors is processed in real time through edge computing, and the pre-processed results are then uploaded to the cloud for fusion analysis, providing decision support and emergency response information to city managers [229].

Overall, by leveraging high-precision fused positioning, low-latency collaborative communication, and multi-modal real-time monitoring, the IoT sensor network lays the foundation for UAM systems to achieve safe and efficient low-altitude operations within complex urban environments [230].

*AI as Predictive Conflict Resolution and Autonomous Control Layer.* In the management of UAM, AI undertakes the core responsibility of shifting the paradigm from passive reaction to proactive prediction and autonomous control.

Firstly, an Operational Digital Twin (ODT), fed by real-time data from IoT sensors, constructs a high-fidelity virtual mirror of the operating environment. On this basis, an AI engine performs simulations and forward-looking analysis to effectively predict potential flight conflicts and generate optimal avoidance strategies, thereby preventing safety incidents from occurring in the physical world [231]. Secondly, neural network-based models for autonomous diagnostics and flight control continuously monitor a UAV’s operational parameters. This allows them to diagnose potential faults in advance and trigger early warnings, significantly enhancing the aircraft’s reliability and autonomy [232]. Finally, DL-driven object recognition algorithms, utilizing optical and multispectral sensors onboard UAVs, enable the real-time detection and classification of ground-based targets such as pedestrians and vehicles. These algorithms can maintain high accuracy even under complex lighting conditions, providing critical support for air traffic command and public safety decision-making [233].

*Blockchain as Secure and Auditable Governance Layer.* Through its decentralized, cryptographically secure, and immutable characteristics, blockchain technology provides a cyberattack-resistant and trustworthy distributed ledger for UAM, designed to support governance, auditing, and incident investigation. In the domain of UTM, lightweight permissioned blockchain solutions like UTM-Chain, based on platforms such as Hyperledger Fabric, have been proposed. These systems are used to record aircraft registration information, approved flight plans, real-time position data, and communication logs, ensuring the security and immutability of all traffic data [19]. Furthermore, blockchain offers a DID management and access control solution for participants including aircraft, operators, and regulatory bodies. Smart contracts can automatically execute pre-defined access policies, ensuring that sensitive surveillance data is only accessed by authorized parties, thereby preserving privacy and data sovereignty [234].

This architecture reflects a profound shift from traditional, centralized control to decentralized governance. Whereas conventional air traffic control relies on a single central authority, a blockchain-based UAM traffic management system distributes the ledger of flight plans and operational data across the nodes of all stakeholders. Trust is no longer derived from a central entity but is instead achieved through cryptographic consensus across the network [19]. This model not only greatly enhances the system’s resilience and resistance to attacks but also lays the foundation for building a more open, equitable, and scalable future air traffic ecosystem.

### 4.3. Precision Agriculture

Precision agriculture represents another key domain for the application of the integrated infrastructure. By introducing data-driven decision-making into agricultural production, this technological framework is poised to revolutionize traditional farming practices, enabling both the efficient utilization of resources and enhanced transparency throughout the food supply chain.

#### 4.3.1. Problem Statement: Inefficiency, Uncertainty and Opacity in Agricultural Production

Traditional agriculture has faced three core challenges: low resource-utilization efficiency, uncertainty in the production process, and a lack of supply chain transparency. Firstly, a reliance on extensive irrigation and fertilization methods leads to significant waste of water and fertilizer in the field. This not only drives up production costs but also has a negative environmental impact on water and soil quality [6,235,236]. Secondly, factors such as the sudden outbreak of pests and diseases [85,237,238], extreme weather events [239], and slow changes in the physicochemical properties of soil [240] cause sharp fluctuations in crop yield and quality. Farmers often make decisions based on experience, lack precise and data-driven guidance, making it difficult to respond effectively to rapidly changing field conditions. Finally, within the long supply chain from farm to end-consumer, information silos and insufficient transparency lead to frequent food fraud. This severely erodes consumer trust in food safety and traceability [241]. These three interconnected problems constrain the development of modern agriculture towards a more intelligent and sustainable future.

#### 4.3.2. Integrated Solutions: From Data-Driven Fields to Transparent Tables

**Figure 7:**
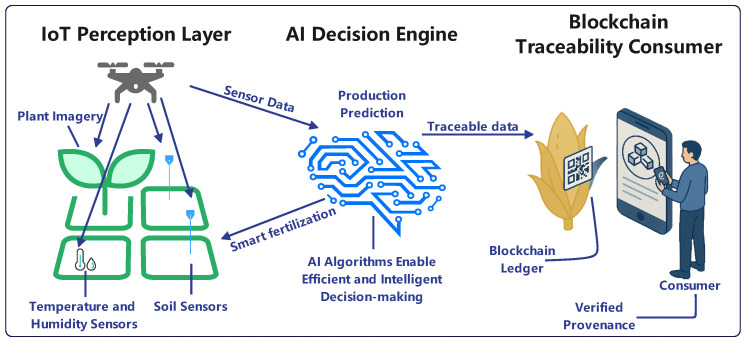
The integrated architecture for precision agriculture. The IoT Perception Layer uses aerial and soil sensors to gather farm data. The AI Decision Engine processes this data for tasks like production prediction and smart fertilization. The Blockchain Traceability layer provides consumers with a verified, immutable record of the product’s provenance from farm to table.

As shown in Figure 7, the integration of IoT, AI, and blockchain technologies enables a transparent, data-driven, and intelligent agricultural ecosystem, where real-time sensor data supports precise decision-making, AI algorithms optimize production processes, and blockchain ensures traceability and trust across the supply chain.

*IoT as High-resolution Farmland Perception Grid.* IoT sensor networks, by combining in-ground soil sensors with UAV-mounted remote sensing equipment, form a high-precision digital perception grid covering entire fields. This grid provides real-time data support for precision agriculture. On the ground, integrated, multi-parameter sensors for soil moisture and NPK (nitrogen, phosphorus, potassium) content can continuously monitor water and fertilizer status, providing an evidentiary basis for irrigation and fertilization decisions [242]. From the air, UAVs equipped with multispectral and hyperspectral cameras can rapidly acquire large-scale crop spectral reflectance data during flight. Through vegetation index models such as the Normalized Difference Vegetation Index (NDVI), these platforms can accurately assess crop health, chlorophyll content, water stress, and nutritional status. This effectively supports the early diagnosis of pests and diseases as well as the monitoring of growth dynamics [79].

When combined with edge computing and cloud-based analytics, this high-resolution perception grid enables real-time alerts and fine-grained management, significantly enhancing both resource-utilization efficiency and the overall intelligence of farm management.

*AI as Agronomic Intelligent Decision-making Engine.* By processing and analyzing the multi-modal data collected by IoT sensor networks, AI algorithms enable efficient, intelligent decision-making in precision agriculture. Firstly, smart diagnostic models based on DL and CV can perform real-time analysis of high-resolution images captured by UAVs. This allows for the automatic identification of early-stage symptoms of pests and diseases, as well as areas of crop nutrient deficiency, with detection speeds and accuracy rates that significantly surpass traditional manual inspection methods [57,84,237]. Secondly, by combining historical crop growth data with real-time perception data, ML models can make reliable predictions about future yields. This capability assists farmers in developing scientific planting and management plans [243].

In the domain of variable-rate precision operations, an AI engine analyzes differences in crop growth across various zones to generate a spatially distributed prescription map. This map then guides UAVs or intelligent ground-based agricultural machinery to perform differentiated spraying and fertilization. For these AI-generated decisions to be physically effective, the models must also account for the complex physics of the downwash flow field from the UAV rotors. This airflow is critical as it is significantly altered by confined structures like greenhouses [244] and interacts directly with the crop canopy [245], which ultimately governs the deposition concentration, uniformity, and effective range of spraying operations [246]. Based on the prescription map, this technology enables the application of higher doses to specific target areas while reducing or ceasing the use of chemicals and fertilizers in healthy zones. This approach maximizes resource-utilization efficiency and reduces environmental pollution [246,247]. This intelligent decision-making framework, which combines the IoT with AI, not only increases the level of precision in agricultural production but also provides crucial technical support for sustainable development.

*Blockchain as Traceable Ledger “From Farm to Fork”.* By providing a shared and immutable distributed ledger, blockchain technology enables full transparency and traceability for the agricultural product supply chain. Consumers can simply scan a QR code on the packaging to access a product’s complete life history, which effectively combats food fraud and enhances trust [241]. To address the challenge of storing large-scale, unstructured agricultural data, research by Santhiya et al. has proposed a dual storage model utilizing blockchain + IPFS. Under this model, massive raw data, such as high-definition images and sensor time-series data, is stored on the decentralized InterPlanetary File System (IPFS) network. Only the cryptographic hash of this data is recorded on the blockchain. This approach ensures data integrity and immutability while avoiding blockchain storage bottlenecks, thereby achieving a balance between security and scalability [248]. Furthermore, blockchain can be used to record and convey value-added information, such as organic certifications, as well as environmental contributions like the amount of water saved during production. This enables quantifiable, trustworthy certification and the effective communication of brand value [241,249].

This integrated architecture reveals a symbiotic data relationship between on-farm operations and the post-farm supply chain. Within this framework, the data from the IoT and AI used to optimize on-farm activities flows throughout the entire supply chain via the trusted medium of the blockchain. Ultimately, the consumer receives not just a physical product, but also a verifiable digital archive of its origin and quality. This creates a new dimension of value for premium agricultural products and provides a powerful economic incentive for farmers to adopt advanced technologies.

### 4.4. Other Scenarios

Beyond the primary application domains discussed above, the integrated infrastructure composed of the IoT, AI, and blockchain also demonstrates broad applicability across several other emerging scenarios. This further underscores its potential as a general-purpose enabling platform.

#### 4.4.1. Problem Statement: Lack of Environmental Data and Limitations of the Travel Experience

Societal development faces multifaceted challenges, including the need for real-time monitoring of the urban environment and the desire to create smarter, more immersive cultural and tourism experiences for residents and visitors within the context of smart cities. Low-altitude technology holds immense potential in these domains.

#### 4.4.2. Integrated Solution: A Common Platform for Monitoring and Experience

The synergistic integration of the IoT, AI, and blockchain technologies constructs a highly flexible, general-purpose platform. Through adaptive adjustments, this platform can simultaneously serve both the public service sector and the commercial entertainment sector [21,250]. In environmental monitoring applications, UAVs equipped with specialized IoT sensors can rapidly reach areas that are difficult for humans to access, enabling them to collect real-time environmental data and generate high-resolution urban pollution maps. This data is transmitted via IoT protocols to a central database for processing, providing continuous updates on environmental conditions [251]. AI algorithms then conduct in-depth analysis of the collected data, making it possible not only to trace pollution sources but also to predict pollution diffusion trends, thereby providing a scientific basis for environmental decision-making. Finally, all environmental data is recorded on a blockchain, forming a tamper-resistant and publicly verifiable environmental ledger. This provides a solid foundation for environmental law enforcement, policy evaluation, and public trust [252,253].

In the domain of low-altitude tourism and entertainment, the integrated system offers visitors scenic aerial routes, immersive aerial photography experiences, and even future “air taxi” commuting services [254]. Within these applications, AI is responsible for intelligently planning flight paths, managing passenger flow, and ensuring flight safety [255], IoT provides immersive data streams to enhance the user experience [256], blockchain enables secure and automated ticketing, identity verification, and access control through smart contracts, thereby guaranteeing the transparency and security of the system’s operation [257].

#### 4.4.3. The Economic Model of Infrastructure-as-a-Service (IaaS)

Constructing a city-wide intelligent low-altitude infrastructure requires enormous upfront investment, making it difficult for any single application to sufficiently amortize the costs and achieve a return on investment [258]. To enhance economic viability, the IaaS model has been proposed. Under this model, a government body, a large corporation, or a public–private partnership is responsible for building and operating the underlying IoT perception network, AI computing platform, and blockchain trust architecture. Upper-layer application providers, such as logistics companies, tourism operators, and municipal departments, can then access this infrastructure on a “plug-and-play” basis via standardized API interfaces and pay on a per-use or subscription basis [258,259]. This model not only effectively amortizes construction and operational costs but also promotes multi-party innovation through an open platform, giving rise to novel services like logistics dispatching, environmental monitoring, and low-altitude tourism [260]. At the same time, the IaaS model offers a new paradigm for urban planning and digital economy development, fostering collaboration between public and private sectors to jointly create a safe, efficient, and sustainable low-altitude intelligent ecosystem [261].

### 4.5. Summary

This section demonstrates the practical value of integrating IoT, AI, and blockchain technologies across typical low-altitude application scenarios. In urban logistics, they enable real-time tracking, intelligent route optimization, and verifiable delivery through smart contracts. For urban air mobility and surveillance, IoT ensures situational awareness, AI supports predictive control and fault diagnosis, while blockchain secures traffic data and enables decentralized airspace governance. In precision agriculture, UAV-based sensing and AI analytics improve crop health monitoring and yield forecasting, and blockchain ensures traceability across the food supply chain. Additional use cases, such as environmental monitoring and aerial tourism, highlight the flexibility of this integrated architecture. The proposed Infrastructure-as-a-Service (IaaS) model further enhances scalability and economic feasibility by enabling shared access to core infrastructure across sectors. Overall, these scenarios reflect the broad applicability and transformative potential of the IoT–AI–blockchain synergy in building safe, intelligent, and trustworthy low-altitude ecosystems.

## 5. Challenges and Future Directions

**Figure 8:**
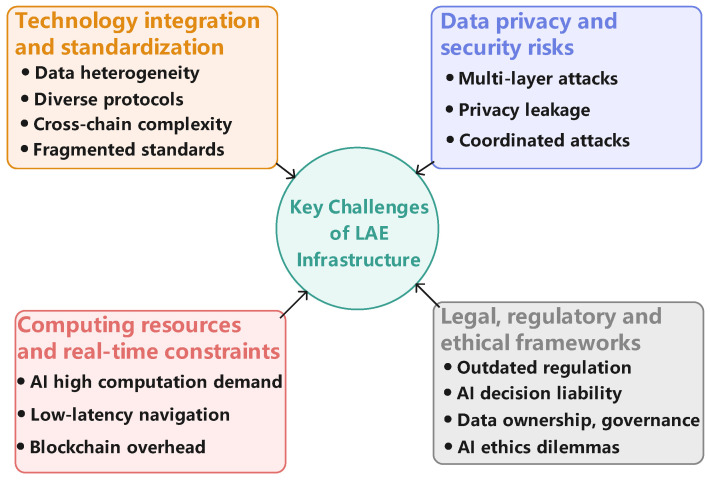
Key challenges of LAE infrastructure.

Although the synergistic integration of the IoT, AI, and blockchain paints a compelling vision for a safe and intelligent LAE infrastructure, its large-scale deployment and widespread application still face a series of profound and complex challenges. These challenges are not merely technical but are also deeply intertwined with multiple dimensions, including data security, system performance, and legal and ethical considerations. Collectively, they form the key agenda for future research and development in this field. This section aims to conduct a critical analysis of these core obstacles and propose forward-looking directions for future research. Figure 8 illustrates four specific challenges presented in this section.

### 5.1. Technology Integration and Standardization

The construction of LAE infrastructure is not a from-scratch endeavor but requires the integration of existing technologies from diverse domains that adhere to different standards. Therefore, the primary challenge is not an absence of standards but the fragmentation and conflict among them, which creates system-level interoperability barriers. Consequently, the focus of future research should shift from pursuing a single, monolithic standard to developing intelligent gateways and middleware. These technologies should be capable of bridging disparate standards and achieving semantic interoperability.

#### 5.1.1. Challenges

The development of LAE infrastructure confronts significant interoperability barriers, which primarily manifest in three areas: data format heterogeneity, diversity in communication protocols, and the complexities of cross-chain communication. Crucially, the challenge of data heterogeneity extends beyond mere format conversion; it directly impacts the core value propositions of the proposed architecture: trust and intelligence. In addition, the challenge of interoperability is most acute at the interface between the IoT Perception Layer and the Blockchain Trust Layer, creating fundamental tensions that affect the integrity of the entire system.

At the data level, the diversified sensors onboard UAVs generate data in a wide variety of formats [262]. This includes aerial imagery in GeoTIFF format, point cloud data in LAS/LAZ formats, and 3D models in formats such as OBJ or FBX [263,264]. Not only do these data structures differ, but their metadata definitions and embedding methods also lack a unified standard. This presents formidable challenges for the fusion and analysis of multi-source data [265,266]. Low-quality data severely undermine the reliability of both AI and blockchain systems. For AI models, heterogeneous and unstandardized inputs trigger the “Garbage In, Garbage Out” effect, resulting in faulty predictions and unsafe decisions. Likewise, while blockchain guarantees data immutability, it cannot ensure data integrity—permanently recording unreliable information that ultimately erodes the very trust it is meant to establish [267]. Moreover, storing massive, continuous, and often unstructured IoT data streams directly on-chain is infeasible due to excessive cost, latency, and storage limitations (i.e., “blockchain bloat”) [162]. Therefore, raw data must be abstracted, aggregated, or hashed before being recorded—a necessary but vulnerable preprocessing step [268]. The transformation of rich, contextual IoT data into compact blockchain-compatible representations can create a “semantic gap”, where essential meaning or situational context is lost [269]. In this sense, blockchain immutability merely preserves the form of data, not its fidelity in representing the original physical event, reflecting an extended manifestation of the “Garbage In, Garbage Out” problem.

At the communication level, communication between UAVs and GCS, as well as intra-swarm communication, may utilize different protocols [270]. This creates a multi-language environment that hinders seamless coordination between devices from different manufacturers and with varying functionalities. This issue is magnified at the IoT-blockchain boundary. IoT devices typically rely on lightweight, low-power protocols like MQTT or CoAP, which are ill-suited for the resource-intensive peer-to-peer (P2P) gossip protocols used by blockchain nodes for consensus and state synchronization [271]. A resource-constrained sensor cannot realistically run a full blockchain node or even a light client. This protocol mismatch necessitates the use of intermediaries, such as gateways or oracles, to bridge the two ecosystems [272]. However, these intermediaries introduce a critical trade-off: they solve a technical problem but reintroduce a degree of centralization. If a gateway is compromised, it can feed malicious or erroneous data to the blockchain, fundamentally undermining the decentralized trust the system aims to create [273]. This “gateway dilemma” means the system’s security is no longer solely dependent on the robustness of the blockchain’s consensus but also on the security of these centralized or semi-centralized bridging components [267]. This shifts the trust assumption from the chain itself to the entire pre-chain data pipeline. For AI models that rely on blockchain for trusted data, this means that the integrity of their decisions is contingent on the reliability of this fragile interoperability layer.

At the blockchain level, multiple participants may operate independent blockchain platforms. Achieving trusted data exchange between these heterogeneous chains represents a significant technical challenge [274,275]. Although existing solutions such as Notary schemes or Relay chains offer potential pathways, they often introduce new centralization risks and struggle to meet the LAE’s stringent requirements for high efficiency, decentralization, and security [276,277]. Collectively, these interoperability barriers constitute a critical challenge for the development of intelligent low-altitude infrastructure, necessitating innovative solutions to achieve true system integration.

#### 5.1.2. Future Directions

Future research should pivot from pursuing a single, monolithic standard to building “bridge” technologies that connect disparate standards in order to achieve true interoperability for LAE infrastructure. Firstly, a semantic interoperability data model based on shared ontology should be developed. This involves designing a unified semantic model for core concepts such as aircraft, airspace, and missions, and creating adaptive mappers to automatically translate multi-source data into this common model [278]. Secondly, AI-driven adaptive gateways use ML to automatically identify and convert heterogeneous protocols and enable dynamic protocol translation and data formatting, thus avoiding the limitations of hard-coded logic [279]. Thirdly, it is necessary to build a cross-standard collaborative middleware framework, integrating standards from bodies like the IEEE in a plug-in-based manner, and decouple upper-layer applications from underlying standards, enabling true plug-and-play integration [280]. Fourthly, to ensure data integrity at the IoT–Blockchain interface, a standardized and lightweight IoT message format for LAE is essential. This standard would mitigate data heterogeneity while embedding security and blockchain compatibility at the source. A canonical JSON message should include key fields such as DeviceID (DID for verifiable origin), Timestamp (UTC precision), MessageType (e.g., telemetry, event), Payload (structured data), and Signature (cryptographic proof). The serialized message can be hashed (e.g., SHA-256) for compact, immutable on-chain anchoring, preserving integrity without storage overhead. Combined device and blockchain timestamps form a verifiable audit trail, while the signature ensures non-repudiation. Finally, drawing inspiration from the core mechanism of blockchain cross-chain protocol to design decentralized, cross-standard trust protocols. This would address the challenges of establishing trust and interoperability between different technological ecosystems [281]. These research directions will lay a solid foundation for the construction of an efficient, secure, and intelligent low-altitude infrastructure.

### 5.2. Data Privacy and Security Risks

The fusion of the IoT, AI, and blockchain, while enhancing system capabilities, simultaneously creates an unprecedentedly complex attack surface. The security challenge has evolved from traditional network perimeter defense to a multi-layered, distributed security problem that spans the entire perceive-decide-act chain and involves data, algorithms, and physical entities. This paradigm shift demands that security strategy transition from perimeter defense to a model of endogenous immunity, where security capabilities are intrinsically embedded within every component and interaction of the system. Consequently, future research must shift its focus from passive defense to proactive immunity, prioritizing the development of endogenous security mechanisms such as privacy-preserving computation and distributed trust management.

#### 5.2.1. Challenges

In the integrated architecture of low-altitude infrastructure, multi-layered attack surfaces interact to increase overall system risk. Firstly, the perception layer is vulnerable to physical or electromagnetic interference. An attacker could tamper with IMU data by injecting electromagnetic noise or could implement GPS spoofing, causing the UAV to deviate from its navigation path. Moreover, resource-constrained IoT terminals have difficulty deploying complex protective measures [282]. Secondly, AI and the decision-making layer face risks from data poisoning and adversarial example attacks, which can induce misjudgments and endanger flight safety [283]. In the network and communication layer, beyond traditional signal eavesdropping and jamming, this layer is susceptible to the leakage and tampering of AI model parameters during transmission, the manipulation of P2P communication between blockchain nodes, and Distributed Denial-of-Service (DDoS) attacks on cloud and edge platforms [284]. Finally, attackers can launch coordinated, cross-layer attacks. For example, they might first use perception-layer spoofing to mislead the AI’s path planning, then exploit blockchain transaction confirmation delays (a timing attack) to block the dispatch of emergency commands. This can ultimately cause a safety incident in the physical world and exacerbate systemic cascading risks [285].

As UAVs equipped with various sensors perform their missions, they inevitably collect large amounts of environmental data, which gives rise to significant privacy risks. Firstly, onboard equipment can capture sensitive information such as facial features, indoor activities, and vehicle trajectories, challenging existing privacy protection regulations [286]. Secondly, if this raw data is leaked, it not only faces the risk of direct exposure but can also be used for secondary inference through AI techniques to build detailed user profiles, further exacerbating the exposure of personal privacy [287]. Finally, although blockchain technology ensures data integrity through its immutability, the transparent nature of public chains could lead to sensitive information being permanently recorded and tracked on-chain, which would further expand the scope of potential privacy breaches [288].

#### 5.2.2. Future Directions

To counter physical layer attacks, future research should focus on developing resilient navigation algorithms based on multi-modal sensor data fusion. This will ensure the reliability of a UAV’s positioning even when GPS signals are jammed or spoofed [289]. Secondly, to defend against attacks targeting AI models, research is needed into security-enhanced Federated Learning (FL) frameworks. These frameworks should employ techniques such as differential privacy and adversarial training to enhance a model’s resilience to data poisoning and adversarial examples while simultaneously protecting data privacy [290]. Furthermore, to address privacy leakage from data collection, a combination of Zero-Knowledge Proofs (ZKPs) and permissioned blockchains can be utilized. This approach allows for the verification of task compliance without exposing raw data, thereby achieving secure on-chain data governance [291]. Finally, to defend against cross-layer coordinated attacks, a proactive defense framework based on Digital Twins should be constructed. By simulating and predicting attacks in a virtual space, such a framework would enable a strategic shift from passive reaction to proactive, predictive defense.

### 5.3. Computing Resources and Real-Time Constraints

In practice, LAE infrastructure confronts a severe performance trilemma: the difficulty of simultaneously satisfying the high computational throughput required by AI algorithms, the sub-second real-time decision-making demanded for flight safety, and the high consensus overhead necessary for blockchain to ensure trust. These three factors are mutually constraining and form the core bottleneck for system scalability. The future breakthrough lies in a combined strategy of computation offloading and algorithmic lightweighting to achieve the optimal allocation and layered decoupling of computational tasks across the cloud, the edge, and end-devices.

#### 5.3.1. Challenges

In low-altitude environments, deploying advanced AI models like large-scale ViTs for high-resolution image analysis or complex MARL policies for swarm coordination necessitates processing large-scale, multi-source data streams, with their high parameter counts placing extreme demands on computational resources. Concurrently, safety-critical functions such as onboard YOLO-based collision avoidance and RL-driven route replanning require decisions at the millisecond or sub-second level, posing a stringent real-time performance challenge [292]. Furthermore, while blockchain provides decentralized trust, conventional consensus algorithms, particularly the energy-intensive Proof-of-Work (PoW), fail to meet these real-time demands due to low Transactions Per Second (TPS) and high confirmation latency. Even efficient algorithms like PBFT introduce non-negligible delays from their multiple rounds of communication and verification, reflecting the well-known scalability trilemma of simultaneously achieving decentralization, security, and scalability [293].

#### 5.3.2. Future Directions

To resolve the trilemma of AI, real-time performance, and blockchain, researchers have proposed an overall strategy of layered decoupling, with energy efficiency as a central design principle. The first approach involves pushing computation down to edge nodes—such as UAVs, ground stations, or 5G base stations—to reduce decision latency and network bandwidth consumption [294]. However, as edge devices are resource-constrained, this necessitates combining model compression techniques with efficient network architectures to reduce model size while maintaining performance. It also involves exploring cloud-edge-device collaborative inference to achieve dynamic, cross-layer task scheduling and optimal global resource allocation [295]. Secondly, to address the computational limitations and network instability of IoT nodes, lightweight consensus algorithms must be designed. For example, improved Byzantine Fault Tolerance (BFT) protocols are expected to enhance system throughput and robustness [296]. Finally, specialized hardware, including GPUs, reconfigurable FPGAs and Vision Processing Units (VPUs), can achieve a complementary match between software and hardware characteristics through algorithm-hardware co-design [297]. Furthermore, Chiplet technology offers a new paradigm for building modular and scalable onboard computing platforms for UAVs, laying a solid foundation for the efficient and secure operation of intelligent low-altitude infrastructure [298].

### 5.4. Legal, Regulatory and Ethical Frameworks

The pace of technological development has far outpaced the evolution of legal, regulatory, and ethical norms, leaving the LAE in a state of “regulatory vacuum” or “regulatory lag”. As AI begins to make autonomous decisions that can impact safety in the physical world and affect individual rights, traditional, human-centric frameworks for liability attribution and ethical review risk becoming obsolete. Therefore, constructing an adaptive and forward-looking governance system is a fundamental prerequisite for ensuring the sound development of the LAE.

#### 5.4.1. Challenges

Amidst the rapid development of low-altitude infrastructure, existing aviation regulations are struggling to adapt to emerging operational models such as AI-driven high-autonomy, BVLOS, and swarm operations. The U.S. Federal Aviation Administration’s (FAA) Part 107 rules, for instance, do not yet provide specific certification and operational standards for autonomous flight systems [299]. Similarly, while the European Union Aviation Safety Agency (EASA) is advancing UAV management through its U-space framework, legal support for AI-driven decision-making and swarm collaboration remains insufficient [300]. Furthermore, the regulation of the LAE spans multiple government departments, where ambiguous divisions of responsibility can lead to regulatory fragmentation and policy conflicts. Compounding this complexity, regulations concerning BVLOS permits, safety assessments, and data privacy are inconsistent across different countries and regions. This creates significant compliance challenges for cross-border operations and constrains the development of international UAV services and collaborative networks.

In the event of a UAV accident caused by an autonomous AI decision, it is exceedingly difficult to apportion liability among the algorithm developer, data provider, operator, and owner. The black box nature of DL models renders the decision-making process opaque, which further complicates post hoc accountability [301]. Additionally, the massive datasets generated by low-altitude platforms hold high commercial value, yet the rights of ownership, usage, and revenue remain poorly defined, leading to conflicts of interest among multiple parties [302]. Meanwhile, although Decentralized Autonomous Organizations (DAOs) could support cross-domain data collaboration, their legal status is ambiguous, and members are often treated as having unlimited joint and several liability, which discourages participation. Moreover, AI systems may face ethical dilemmas analogous to the trolley problem in emergencies; if the decision-making process lacks transparency, it becomes difficult to review and assess its reasonableness [303]. Biases latent in training data could also lead an AI to make discriminatory decisions in obstacle avoidance or path planning, potentially harming the interests of vulnerable groups [304]. As the autonomy of AI increases, the boundaries of human oversight and control become blurred, and existing legal and regulatory frameworks struggle to effectively constrain algorithmic behavior.

#### 5.4.2. Future Directions

As the IoT, AI, and blockchain technologies rapidly evolve within intelligent low-altitude infrastructure, traditional static regulatory models can no longer meet the safety and innovation demands of emerging applications. Thus, the concept of adaptive governance must be introduced to construct a flexible regulatory system capable of evolving in tandem with technology. On one hand, by establishing regulatory sandboxes, authorities can allow companies to test new technologies in a controlled environment. This provides real-world data to support evidence-based rulemaking, thereby shortening the time lag between technological maturation and the publication of official regulations [305]. On the other hand, future governance must achieve a synergy between technology, law, and ethics, utilizing blockchain smart contracts to automatically enforce compliance and implementing the principle of code is law to prevent rules from being circumvented by human actors. Using legal frameworks to clearly define the boundaries for all participants regarding data security, privacy protection, and accident liability. Employing ethical review mechanisms to ensure that technological innovation aligns with core societal values and avoids algorithmic bias and ethical risks. This comprehensive framework will not only enhance regulatory efficiency but also promote the healthy and orderly development of the LAE while safeguarding security.

## 6. Conclusions

The LAE is poised to reshape urban and rural landscapes, offering unprecedented opportunities for innovation in transportation, logistics, agriculture, and a myriad of other sectors. However, its successful and sustainable development hinges on overcoming significant challenges related to security, operational intelligence, and the establishment of trust among all participants. This review has argued that the synergistic integration of IoT networks, AI decision-making, and blockchain-based trust mechanisms provides a powerful and holistic technological foundation to address these challenges effectively.

By leveraging IoT for pervasive sensing and connectivity, AI for intelligent perception and autonomous control, and blockchain for DID, immutable auditability, and automated trusted services, a new generation of secure and intelligent low-altitude infrastructure can be realized. The proposed layered architecture offers a conceptual blueprint for organizing these complex technologies, while the detailed examination of each technological layer and their interplay in various application scenarios underscores the transformative potential of this integrated approach. While significant progress has been made, the journey towards a fully mature and globally adopted LAE requires continued research and innovation across multiple fronts, as outlined in the future research directions. Addressing these challenges will be crucial for unlocking the full economic and societal benefits of the LAE, paving the way for a future where low-altitude airspace is a safe, efficient, and integral part of our daily lives. The continued collaborative efforts of researchers, industry stakeholders, and regulatory bodies will be paramount in navigating this complex but promising technological frontier.

## Figures and Tables

**Table 1:** Comparative Analysis of Integrated Technology Frameworks.

Reference	Technology Synergy	Key Contribution	Scope/Limitations
[19]	IoT + Blockchain	Introduced UTM-Chain for secure UAV traffic management.	Lacks AI for conflict prediction and dynamic control.
[20]	IoT + AI + Blockchain	AI-enhanced blockchain for supply chain transparency.	Not designed for real-time aerial autonomy.
[21]	IoT + AI + Blockchain	Smart city framework improving data security and service intelligence.	Conceptual; lacks safety-critical integration.
[22]	IoT (Crowdsensing) + Blockchain	Blockchain-based crowdsensed UAV traffic data management.	Depends on crowdsensing; limited AI autonomy.

**Table 2:** Comparison of commonly used airborne sensor technologies for low-altitude platforms.

Sensor Type	Sensing Principle	Data Output	LAE Advantage	Limitation	LAE Typical Application
RGB Cameras	Visible light capture	Images, videos	Low cost, intuitive data	Light/weather sensitive, poor penetration	Infrastructure inspections [7,8], precision agriculture [56,57,58,59], accident investigation [9], security surveillance [60]
LiDAR	Measure laser echo time	3D point clouds	High-precision 3D mapping	Expensive	Terrain mapping [25,64,65], infrastructure scan [68], obstacle detection [26,69,70], forestry surveys [72,73], power line safety analysis [66,67]
IR/Thermal Sensors	IR radiation, surface temperature distribution	Thermal image, temperature	Night/low visibility, non-contact temperature measurement	Low image resolution, susceptible to temperature	Night SAR [74,75], overheating fault detection [76], fire point detection [77], agricultural drought monitoring [78,79]
Multispectral Sensors	Multi-band spectral capture	Multi-band images, vegetation index	More information than RGB	Limited spectral resolution	Crop classification, growth and coverage evaluation [56,80,81], pest and disease surveillance [84,85], nutrient non-destructive testing [87]
Hyperspectral Sensors	Continuous narrow-band capture	Hyperspectral Cube	Material composition analysis	Massive data, costly	Environmental monitoring [90], mineral exploration [89]
GNSS/IMU	GNSS positioning, IMU attitude	PVA, timestamp	Provides spatiotemporal reference	GNSS occlusion, IMU drift	UAV autonomous navigation [94]

**Table 3:** Comparison of the progress of low-altitude perception CV models.

Model Type	Segment Type	Key Technical Features	Advantages	Challenges	Application
CNN	YOLO	Single-stage detection	Mature for real-time basic detection	Limited for small, dense targets	Common aerial detection [128]
	Optimized YOLO	Lightweight backbone, attention, multi-scale fusion	Improved small target accuracy	Sensitive to occlusion, background clutter	Small object [129], obstacle detection [113], infrastructure defects [130]
ViT	ViT	Self-attention	Strong in complex scenes	High compute cost, data-hungry	Behavior recognition [131], scene analysis [132]
	Optimized ViT	Occlusion-robust features, early exit, multi-scale fusion	Better under occlusion, blur	Complex, hard real-time deployment	Target tracking [124,125], Object detection [126]
CNN-ViT	BrownViTNet	CNN local features + ViT global modeling	Balanced detail and semantics	Complex, resource intensive	Fine-grained classification [127]

**Table 4:** Overview of RL algorithms for UAV path planning and swarm coordination.

Category	Algorithm	Principle	Advantages	Limitation	Application
RL	DQN/DDQN	Q-value iteration; DDQN mitigates overestimation	Simple, reliable	Poor for continuous	Path planning [34,135,136], obstacle avoidance [146]
	DDPG/TD3	Actor-Critic; TD3 improves stability	Continuous control	Hyperparameter sensitive	Path planning [138,139]
	A3C	Asynchronous Advantage Actor-Critic	Fast convergence, parallelizable	Complex implementation	Navigation [137], multitask [147]
MARL	IQL	Independent Q-learning	Low-cost implementation	Ignores interaction	Simple multi-UAV tasks [148]
	VDN/QMIX	CTDE + value decomposition, QMIXnon-linear mixing	Efficient collaboration	Limited for competitive tasks	Cooperative coverage [141], cooperative transport [143]
	MADDPG	Multi-DDPG + CTDE	Cooperation and competition	Large communication/input overhead	Swarm control [149], communication coverage [150]
	LLM-MARL	LLM-enhanced MARL for high-level reasoning	Fast convergence	High complexity	Enhancing mobile edge computing (MEC) networks [151]

**Table 5:** Emerging applications of LLMs in low-altitude infrastructure management.

Application	Role	Advantages	Challenges	Research
High-level mission planning and autonomous control	Instruction parsing, task planning	Improve task autonomy and environmental adaptability	Logical errors	FLUC [152], LLM-QTRAN [151]
Facilitating advance HMI	Natural language interactions, Intelligent Q&A, Collaborative decision-making	Lower the threshold for operation, enhance trust	Multi-user collaboration complexity	Neuro-LIFT [153], GSCE [155]
Complex decision-making assistment and domain knowledge management	Domain expertise integration, decision-making assisting, what-if analysis	Enhance knowledge intensity	Knowledge base update, intellectual bias	LLMs + RAG to enhance Internet of Drones (IoD) intelligence [156]

**Table 6:** A summary of the use case and integration technology roles.

Scenario	Key Issue	IoT Role	AI Role	Blockchain Role	Reference
Urban logistics and instant delivery	Low last-mile efficiency, delivery verification	Real-time tracking, sensing	Route optimization, resource management	Delivery verification, traceability	[181,182]
UAM and intelligent surveillance	Airspace safety, data security	V2X sensing, positioning	Conflict resolution, diagnostics	UTM, access control	[19,183]
Precision agriculture	Low resource efficiency, opaque supply chain	Multispectral/soil sensing	Pest detection, yield prediction	Full-process traceability, validation	[85,184]
Other Scenarios	Sparse data, single experience	Air quality	Pattern recognition, prediction	Verifiable reports, secure ticketing	[185,186]

## Data Availability

No new data were created or analyzed in this study.
